# Development of a potent embryonic chick lens model for studying congenital cataracts in vivo

**DOI:** 10.1038/s42003-021-01849-0

**Published:** 2021-03-11

**Authors:** Zhen Li, Sumin Gu, Yumeng Quan, Kulandaiappan Varadaraj, Jean X. Jiang

**Affiliations:** 1 Department of Biochemistry and Structural Biology, University of Texas Health Science Center, San Antonio, TX USA; 2 Department of Physiology and Biophysics, Stony Brook University, New York, NY USA

**Keywords:** Biological techniques, Diseases

## Abstract

Congenital cataracts are associated with gene mutations, yet the underlying mechanism remains largely unknown. Here we reported an embryonic chick lens model that closely recapitulates the process of cataract formation. We adopted dominant-negative site mutations that cause congenital cataracts, connexin, Cx50E48K, aquaporin 0, AQP0R33C, αA-crystallin, CRYAA R12C and R54C. The recombinant retroviruses containing these mutants were microinjected into the occlusive lumen of chick lenses at early embryonic development. Cx50E48K expression developed cataracts associated with disorganized nuclei and enlarged extracellular spaces. Expression of AQP0R33C resulted in cortical cataracts, enlarged extracellular spaces and distorted fiber cell organization. αA crystallin mutations distorted lens light transmission and increased crystalline protein aggregation. Together, retroviral expression of congenital mutant genes in embryonic chick lenses closely mimics characteristics of human congenital cataracts. This model will provide an effective, reliable in vivo system to investigate the development and underlying mechanism of cataracts and other genetic diseases.

## Abstract (web summary)

Li et al. report an embryonic chick lens model to study the onset of congenital cataracts. By injecting recombinant retrovirus containing mutants that cause congenital cataract in lumen of embryonic chick lens, they show that their model exhibits cataract lens closely mimicking characteristics of human congenital cataracts.

## Introduction

The description of a congenital cataract is the opacification of all or specific regions of the lens detected at birth or during the first decade of life. According to a meta-analysis, the overall prevalence of congenital cataracts is in the range from 0.63 to 9.74/10,000 (median = 1.71)^1^. Approximately half of the congenital cataracts are characterized as inherited and associated with considerable genetic and phenotypic heterogeneity^2^. Mutations leading to cataract formation have been identified in genes encoding various proteins, such as intracellular lens proteins (crystallins), membrane proteins (connexins (Cx), and aquaporins (AQP0)), cytoskeletal proteins, and transcription factors^3^. To date, 1433 single and recurrent disease-causing sequence variants have been identified (http://cat-map.wustl.edu/) in isolated and syndromic inherited cataracts. Although there are large numbers of mutated genes to be investigated, thus far, genetic knockout or knockin mouse models are the only in vivo models available to study congenital cataracts. The generation of these animal models is known to be cost-ineffective and time-consuming. In addition, it is less feasible to generate a sufficient amount of samples for biological and biochemical analysis. Currently, lens regeneration from endogenous stem cells has been shown to be a potential option for the treatment of cataracts, this method will deploy gene editing using CRISPR/Cas9 technology in order to rectify the genetic mutation in the regenerated lens^4^. When considering these advancements, pharmacological drugs remain the most pragmatic solution to treat cataracts, especially in developing countries where immediacy and cost-effectiveness are key^5^. However, it would be challenging to use animal models as a screening and testing tool to identify potential drug candidates and develop treatment options. There is an unmet need to develop other in vivo model systems.

Embryonic chicks as an animal model to study lens physiology and development have been well established with early studies dating back decades^6–8^, although transgenic approaches in chicken (*Gallus domesticus*) have had limited success. Chicken lens structure and development share a high degree of similarities with the human lens, such as lens accommodation, which does not exist in the rodent lens. To experimentally probe the pathology of cataracts, multiple ex vivo models have been developed with chicken, such as chicken lens ex vivo cultures^9^, lens primary cell^10^, and lens capsule cultures^11^. The RCAS(A) replication-competent avian retrovirus provides a fast and highly efficient system to express exogenous proteins in chick embryos and primary chick cell culture^12,13^. Embryonic lens development offers a unique time window in which in situ retroviral microinjection into the empty lens lumen surrounded by lens cells leads to a specific expression of proteins in the lens^14,15^. This model offers a unique opportunity to express exogenous proteins, especially dominant-negative mutants in the lens with minimal non-specific expression in other tissues as a result of the occlusiveness of the lens lumen. Although promising, the use of this model system to study protein mutations that lead to cataract formation, lens morphological and structural changes, and protein aggregation has not been demonstrated. In this study, through recombinant retroviruses, we express exogenous dominant-negative site mutants of several lens proteins, Cx50, aquaporin 0, and αA-crystallin that cause congenital cataracts in the embryonic chick lens. We show that analogous to human congenital cataracts, these mutants develop lens opacity, altered lens morphology and structure, and increased protein aggregation.

## Results

### Nuclear cataract formation in embryonic chick lenses expressing a site mutant of Cx50

**Fig. 1:**
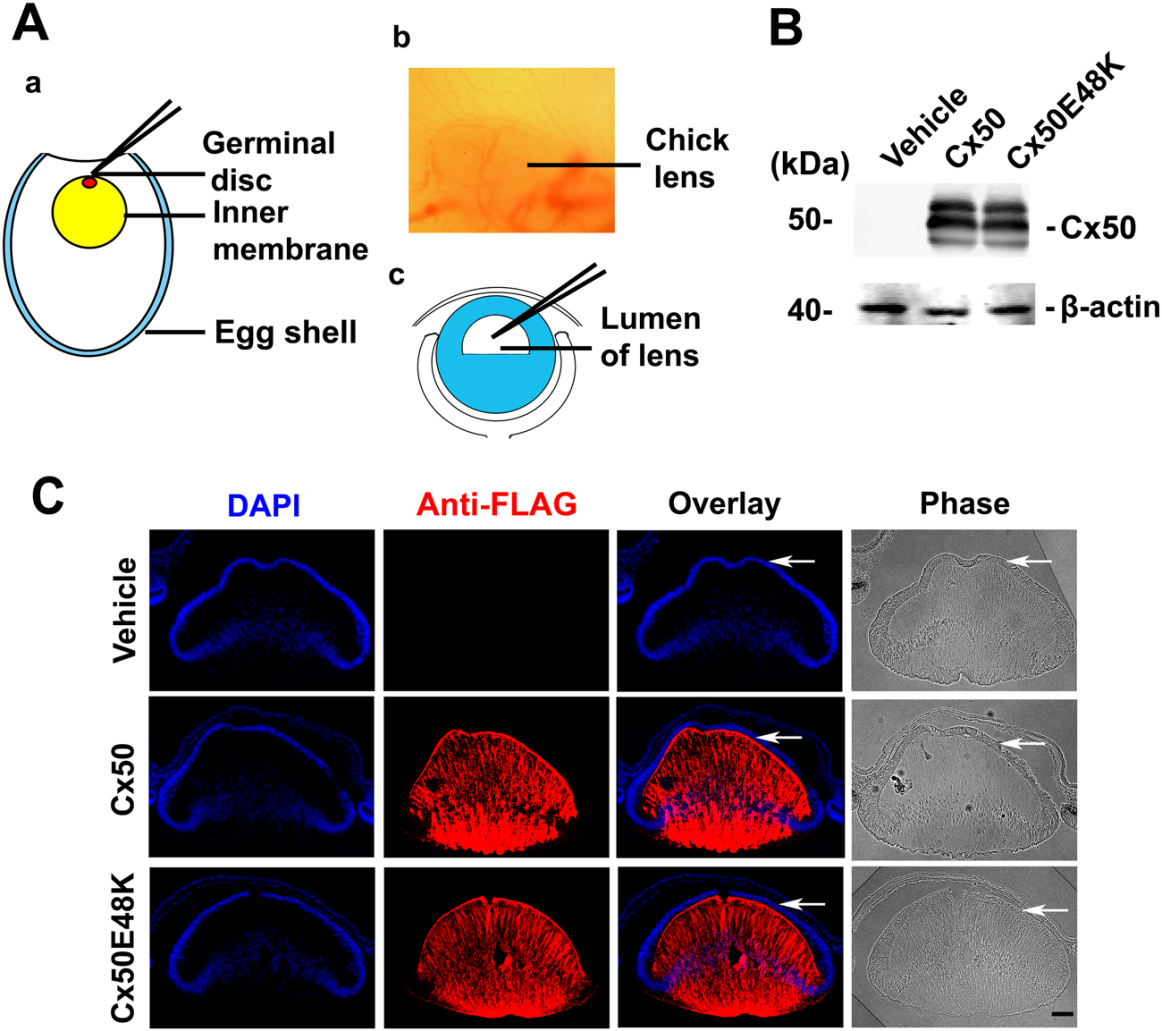
Expression of exogenous connexins in embryonic chick lens in situ. **A** At 68 h of embryonic development (around developmental stage 18), eggshells were incised to reveal live embryos (a and b). Approximately 20 nL solution containing high-titer retrovirus expressing recombinant proteins was microinjected into the lens lumens (c). **B** 48 h after retroviral infection, chick embryonic lenses were collected and crude membrane extracts were prepared. The expression of Cx50 and the Cx50E48K mutation was detected by western blotting using an anti-FLAG tag antibody. **C** 48 h after infection, chick embryonic lenses were collected and immunostained with anti-FLAG antibody and counter-nuclei stained by DAPI. White arrows show epithelial cells at the anterior region of the lens. Bar, 50 µm.

The embryonic development of chick lens provides a unique opportunity to express exogenous proteins in situ exclusively in the lens cells^14^. In this study, we investigated whether the expression of cataract-causing mutants in this embryonic chick system could recapitulate the characteristics of human congenital cataracts. E48K, a site mutation located in the first extracellular loop domain of Cx50, is identified to cause human congenital nuclear cataracts^16^. To achieve exogenous expression exclusively in the embryonic chick lens, we microinjected recombinant retroviruses containing the Cx50E48K mutant at ~68 h of embryonic development (Fig. 1A). Western blot analysis showed comparable expression of wild type (WT) Cx50 and mutant Cx50E48K in the lens (Fig. 1B and Supplementary Fig. 1). By high-resolution immunofluorescence with antibodies against FLAG for detection of exogenously expressed proteins, both WT Cx50 and mutants were primarily localized in lens fiber cells 48 h after infection (Fig. 1C). The majority of the fiber cells continued to express exogenous Cx50 or the mutant 2 days after the injection of recombinant retroviruses.

**Fig. 2:**
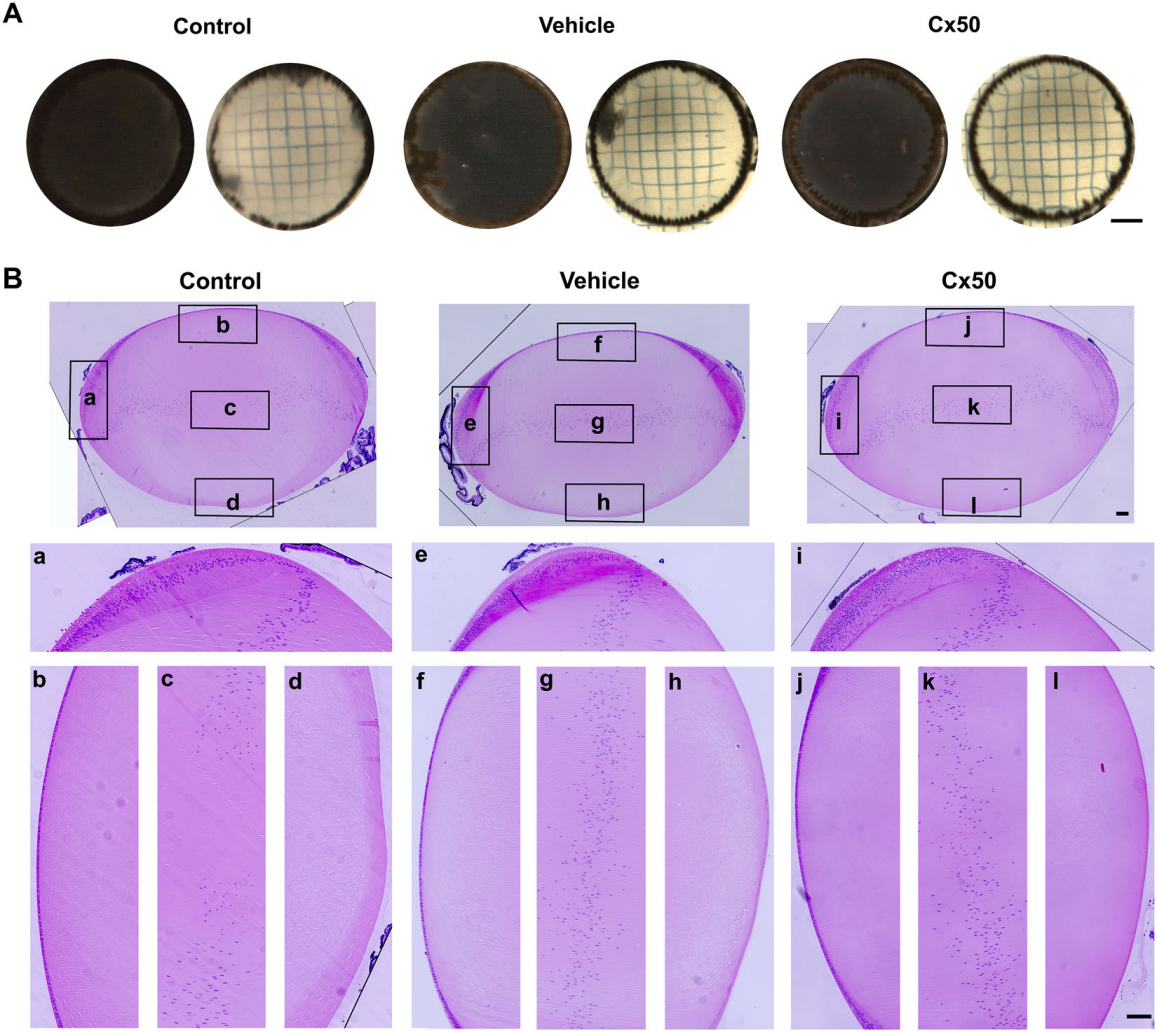
Exogenous expression of WT Cx50 has no effect on lens transparency and structure. **A** Embryonic day 20 lenses with or without injection of recombinant retrovirus containing vehicle or Cx50 were imaged with (right panels) or without grids (left panels). Scale bar: 400 µm. **B** Paraffin lens sections were stained with H&E. Images of midsagittal paraffin tissue sections of lenses injected with a retrovirus containing vehicle or Cx50 were captured with a Keyence BZ-X710 microscope. As indicated by black framed boxes in the entire lens: (a, e, and i) lens equator region; (b, f, and j) lens anterior region; (c, g, and k) lens central region; (d, h, and l) lens posterior region. Scale bar: 50 µm.

**Fig. 3:**
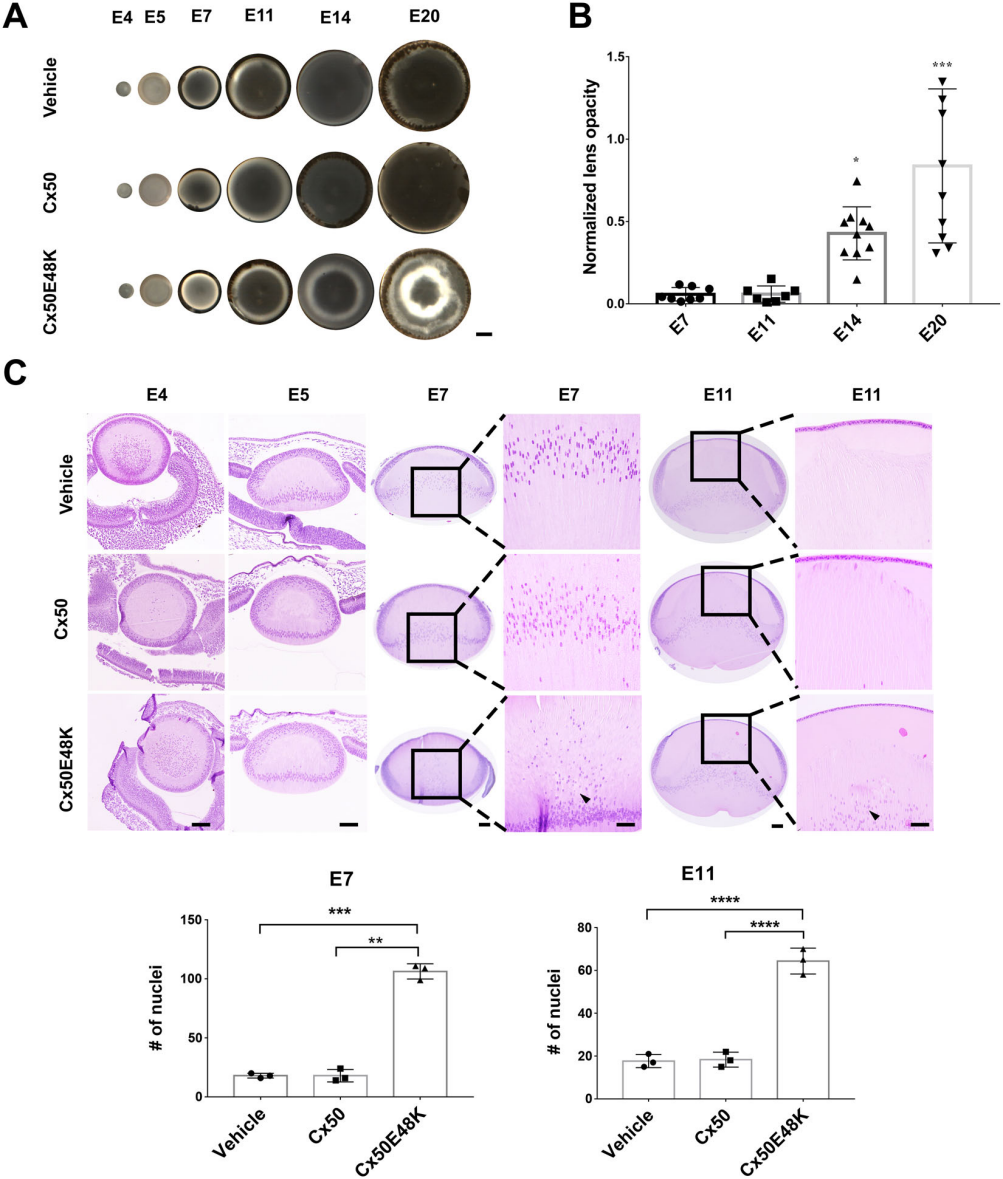
Exogenous expression of the dominant-negative Cx50E48K mutation induces the formation of nuclear cataracts. **A** Optical images of embryonic chick lenses injected with a recombinant retrovirus containing vehicle, Cx50 or Cx50E48K were taken at embryonic day (E), 4, 5, 7, 11, 14, and 20 under identical magnification. Scale bar: 400 µm. **B** The relative magnitude of cataracts was measured and quantified by Image J and normalized with the contralateral lens. The extent of opacity of retrovirus injected lenses was normalized by subtracting that of the contralateral lens and then dividing by that of the contralateral lens. All data are presented as mean ± SEM. *P < 0.05, ***P < 0.001. *n* = 8 for E7, *n* = 7 for E11, *n* = 10 for E14 and E20. **C** Representative images of H&E staining at various stages of embryonic lens development (upper panel). Black arrowhead indicated the abnormal nuclear distribution in Cx50E48K expressing lenses. Scale bar: 50 µm. The nuclei number was counted and the quantification results are shown in the bottom panel. All data are presented as mean ± SEM. **P < 0.01, ***P < 0.001, ****P < 0.0001. *n* = 3.

To control for potential effects of retroviral infection and expression of exogenous proteins in the lens, RCAS(A) retroviral vehicle or recombinant retrovirus containing Cx50 was microinjected into the lens at 68 h after embryonic development. At embryonic day (E) 20, non-injected control lenses and lenses expressing retroviral vehicle and WT Cx50 maintained transparency and light transmission (Fig. 2A). Hematoxylin and eosin (H&E) staining revealed RCAS(A) vehicle and Cx50 infected lenses displayed normal structure when compared with non-injected control lenses (Fig. 2B). In contrast, lenses expressing the Cx50E48K mutant showed nuclear cataract formation at E14, similar to congenital nuclear cataracts that develop in human patients with the Cx50E48K mutation^16^ (Fig. 3A). At early embryonic stages (E4, E5, E7, and E11), a certain degree of lens opacities was observed, but the levels were similar in lenses with or without injection. The appearance of “pseudo” lens cloudiness observed in smaller lenses is caused by light illumination used in capturing the images. This technical issue was mitigated by increasing the lens sizes. At E14 and E20 days, apparent opacities were only shown in lenses expressing Cx50E48K. Quantification analysis showed a significant increase of opacity in E14 and E20 lenses (Fig. 3B). H&E staining of lens sections revealed that structural changes of lenses expressing the E48K mutant appeared earlier than cataract formation (Fig. 3C). At E7, lenses expressing Cx50E48K showed abnormal nuclear localization with a broad and unorganized distribution pattern (Fig. 3C, arrowheads). The abnormal nuclear distribution was continuously observed at E11, nuclei appeared to locate towards the anterior lens region, while nuclei in-vehicle control and WT Cx50 expressing lenses were primarily located around the central region. Quantification analysis showed that numbers of nuclei were significantly increased in E7 and E11 lenses expressing Cx50E48K (Fig. 3C, lower panels). Additionally, at E4, 1 day after the injection, the central region of Cx50E48K injected lenses appeared to have more nuclei than non-injected contralateral control lenses. However, this abnormal phenomenon disappeared in injected lenses at E5, indicating that microinjection manipulation only had a short-term effect before this temporal alteration was recovered.

### Expression of a Cx50 mutant causes heterogeneous lens opacities and a disrupted lens structure

**Fig. 4:**
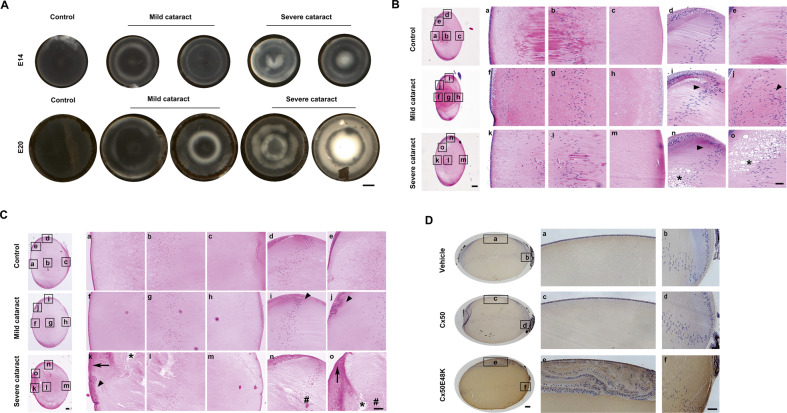
Expression of Cx50E48K mimics characteristics of congenital cataracts with lens opacities, disrupted lens structure, and increased oxidative stress. **A** Lenses were dissected at E14 and E20, and lens opacity was observed under a dissecting microscope. Scale bar: 400 µm. The severity of opacities was categorized as mild and severe cataracts, distinguished by the extent of clarity in the central region of the lens. Morphology and organization of E14 (**B**) and E20 (**C**) chick lenses expressing exogenous Cx50E48K mutation or contralateral lens control were examined by H&E staining. Scale bar for the entire lens: 100 µm. As indicated by black frame boxes in the entire lens, (a, f, and k) anterior lens region; (b, g, and l) central lens region; (c, h, and m) posterior lens region; (d, i, and n) lens equator region. Arrowheads indicate disrupted nuclear distribution. (e, j, and o) showing the most visible changes of the lens. Arrows show liquefied spaces between cortical fibers (evenly red-stained). Asterisks showed enlarged extracellular spaces of lens fibers. Hashtags indicate enlarged fiber cells. Scale bar: 50 µm. **D** Paraffin lens tissue sections of E20 lenses with an injection of recombinant retrovirus containing vehicle, Cx50WT, or mutation were stained with anti-4-HNE antibody by incubation with an avidin-biotin-peroxidase complex (ABC) reagent. Scale bar for the entire lens: 100 µm. Scale bar for partial lens region: 50 µm.

We observed the development of visible lens opacities in all E14 and E20 chick lenses exogenously expressing the Cx50E48K mutant. The extent of lens opacity varied from mild to severe distinguished by the degree of clarity in the central region of the lens (Fig. 4A). During lens development from E14 to E20, the number of severe cataracts increased, and the relative magnitude of cataracts increased by two-fold (Fig. 3B). Very severe nuclear opacities were detected in E20 lenses expressing the E48K mutant (Fig. 4A). The lens structure and nuclear distribution were also altered as shown by H&E staining at E14 (Fig. 4B) and E20 (Fig. 4C). Lenses with relatively mild cataracts at E14 displayed abnormal nuclear localization with a ring-like pattern (Fig. 4B, f and j), while E20 lenses showed enlarged fiber cell morphology in addition to abnormal nucleus distribution (Fig. 4C, f–j). Severe cases of cataracts were often associated with other lesions, such as enlarged extracellular spaces in lens fibers (Fig. 4B, C, k–o). H&E staining of E20 lenses with severe opacities showed apparent structure destruction including enlargement of lens fiber cells (Fig. 4C, hashtags) and increased extracellular spaces (Fig. 4C, asterisks). Additionally, abnormal nucleus distribution (Fig. 4C, arrowheads) and disorganized tissue mass (Fig. 4C, arrow) were found close to epithelial cells. Elevated levels of oxidative stress have been reported during cataract formation^17,18^. The oxidative stress marker 4-hydroxynonenal (4-HNE) was used to estimate the stress in this system. When comparing lenses expressing exogenous WT Cx50 or vehicle control to the Cx5048K mutant, lenses expressing Cx5048K exhibited a strong 4-HNE signal in both epithelial (Fig. 4D, a, c and e) and fiber cells (Fig. 4D, b, d and f).

**Fig. 5:**
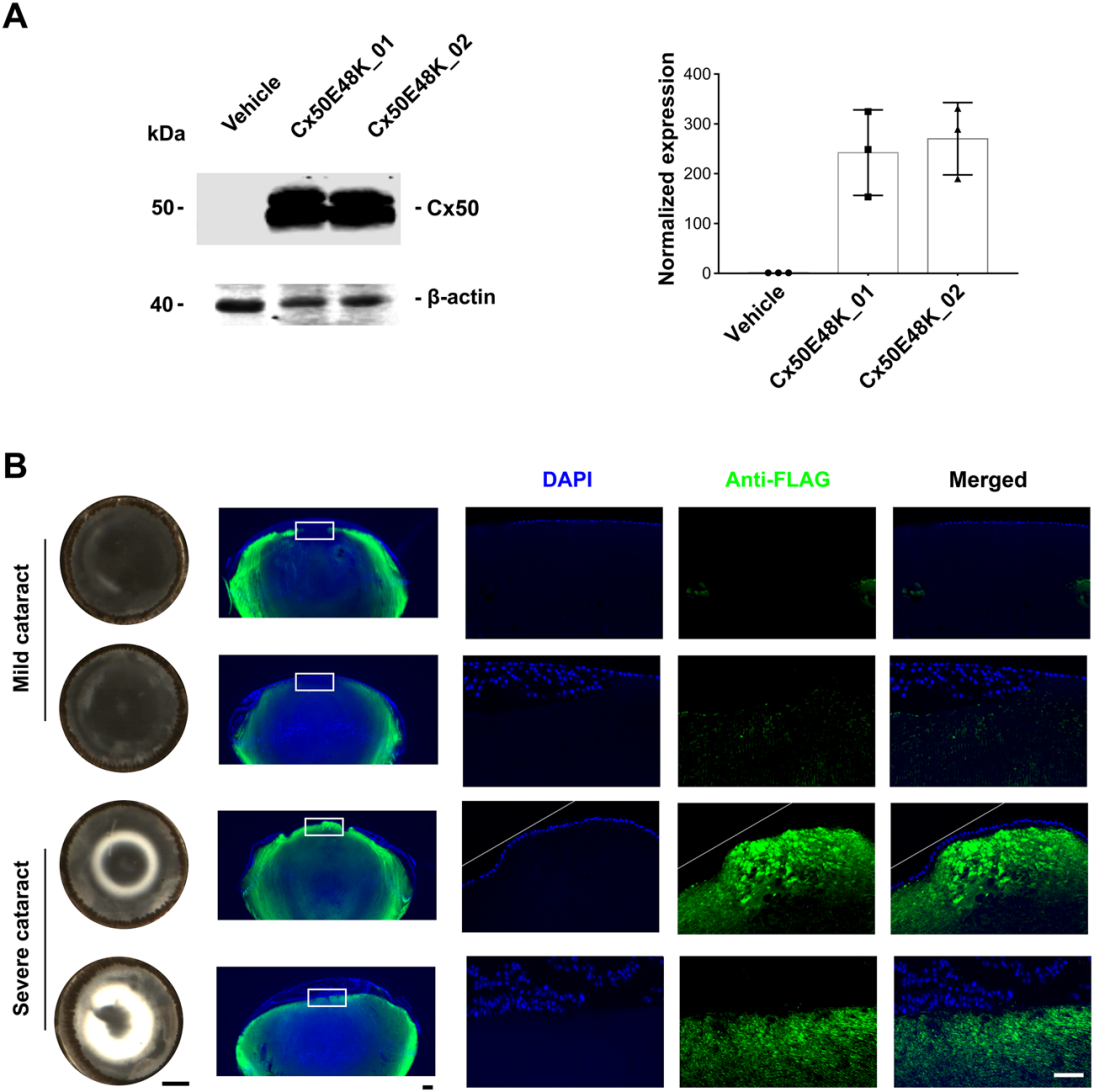
Location of Cx50E48K expression at the anterior lens region defines the severity of lens opacities. **A** Lysates of E14 chick lenses injected with a retrovirus containing vector or Cx50E48K (Cx50E48K_clones 01 and 02, representing lenses forming mild and severe cataracts, respectively) were immunoblotted with anti-FLAG or anti-β-actin antibody. **B** Paraffin tissue sections of E14 chick lenses were immunostained with anti-FLAG or anti-β-actin antibody, and images were captured by light transmission (left panels) and fluorescence (right panels) microscopy. Scale bar for optical images, 400 µm, and for fluorescence images, 50 µm.

To assess the causative relationship between severity of cataracts and expression level and pattern of the E48K mutant, western blot and immunofluorescence were performed in E14 lenses. Quantification analysis of western blot with anti-flag antibody (Fig. 5A, left panel and Supplementary Fig. 2) showed that the amount of Cx50E48K was comparable in embryonic chick lens with both mild and severe opacities (Fig. 5A, right panel). Immunofluorescence also confirmed the comparable expression of Cx50E48K among different lenses. However, in lens samples with severe opacities, more Cx50E48K mutant was localized at the anterior region of lens fibers (Fig. 5B, last two rows of panels). Together, the results suggest that exogenously expressed dominant-negative Cx50E48K mutants result in nuclear cataract formation, abnormal nuclei distribution, distorted lens structures, and increased oxidative stress in embryonic chick lens. Moreover, cataracts formed by Cx50E48K are heterogeneous and this is associated with levels of the mutant expressed at the anterior lens fibers.

### Cortical cataracts and disrupted lens structures in embryonic chick lenses expressing the human AQP0R33C mutant

**Fig. 6:**
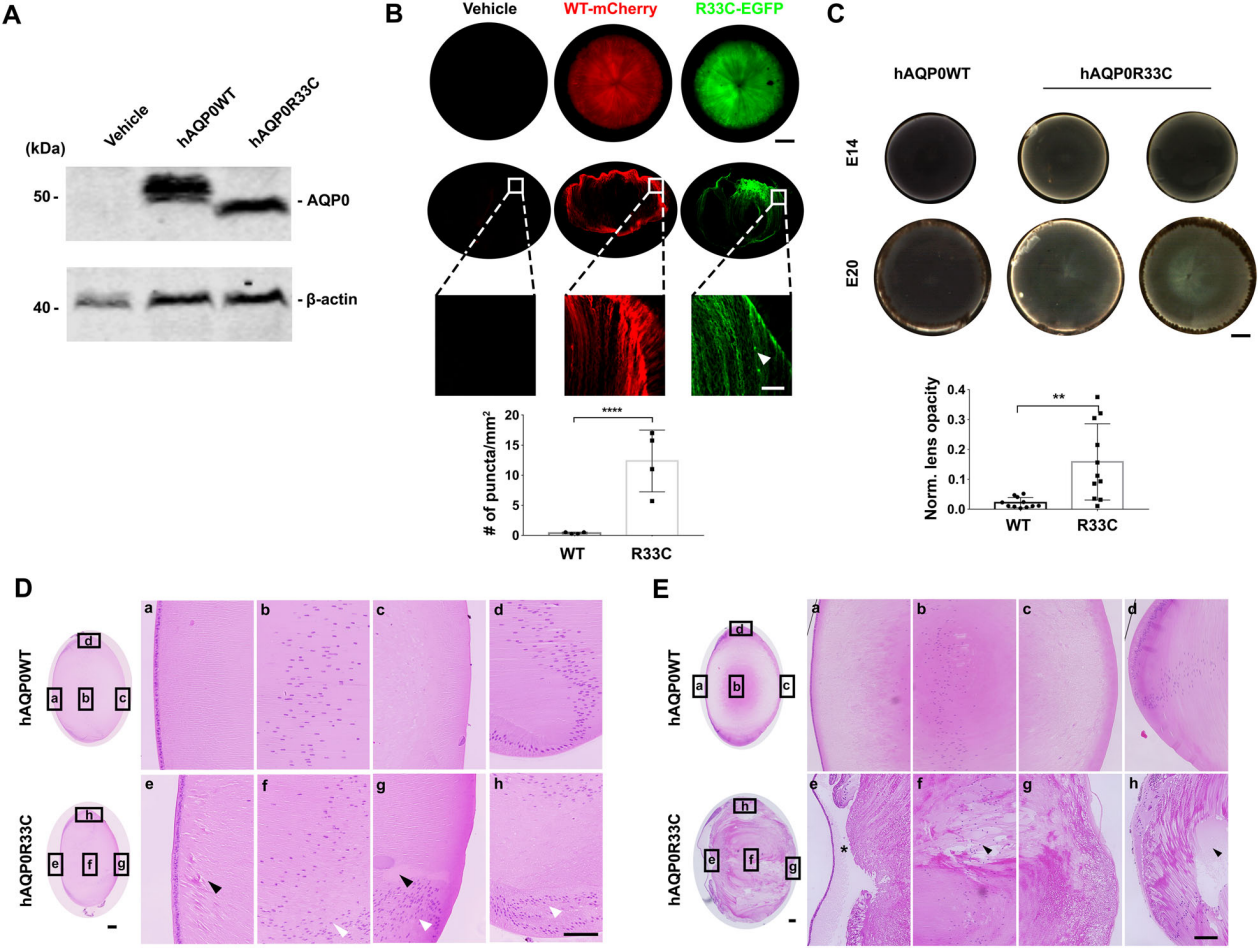
Exogenous expression of the human AQP0R33C mutation induces cortical cataract formation. Embryonic chick lenses were injected with recombinant RCAS(A) expressing WT hAQP0 or the hAQP0R33C at E14 and E20. **A** Lysates of E14 chick lenses were prepared and immunoblotted with anti-AQP0 or anti-β-actin antibody. **B** E20 chick lenses were observed under a fluorescence microscope for AQP0WT-mcherry (red) and AQP0R33C-EGFP (green) expression in the whole lens, respectively (coronal orientations, upper panels). Frozen sections of the same lenses were prepared and fluorescence images were taken (middle panels for the whole lens, and lower panels for partial lens indicated with white box) and puncta numbers were quantified (bottom panel). For each group, *n* = 4. Scale bar: 400 µm for the upper two rows of panels. Scale bar: 50 µm for the bottom panel. **C** Opacity of E14 and E20 chick lenses expressing endogenous hAQP0 or hAQP0R33C mutations were shown by light transmission microscopy (upper panel) and quantified using the same method as described in Fig. 3B (bottom panel). For each group, *n* = 11. Scale bar: 400 µm. Sagittal paraffin sections of E14 (**D**) and E20 (**E**) chick lenses expressing WT hAQP0 or hAQP0R33C mutations were prepared and stained with H&E. Black arrowheads show empty and enlarged extracellular spaces. White arrowheads show abnormal nuclear distribution. Black asterisk points to a detachment region between epithelial and fiber cells. Scale bar, 50 µm. All data are presented as mean ± SEM. **P < 0.01, ****P < 0.0001. *n* = 3.

AQP0 is the most abundant membrane protein in the lens, and AQP0 mutations are commonly associated with human congenital cataracts^19^. To ascertain if this embryonic system recapitulates the characteristics of the AQP0 mutation, as with Cx50, we introduced exogenous WT or the site mutant R33C of human AQP0 into the embryonic chick using microinjection and retroviral infection. The comparable expression of the human AQP0 and R33C mutant was confirmed by western blot with anti-AQP0 antibody (Fig. 6A and Supplementary Fig. 3). The migration of exogenous AQP0 protein and R33C mutant at ~50 kDa was distinct from endogenous AQP0 due to their conjugation with the mCherry and EGFP proteins, respectively. The rich expression of exogenous AQP0 and AQP0R33C mutants were also shown by mCherry (red) and EGFP (green) fluorescence, respectively (Fig. 6B). Interestingly, besides membrane localization, a distinct punctate staining pattern was seen in the R33C expressing lens fiber cells, indicating protein aggregates. Compared to WT expressing lenses, exogenous expression of the human AQP0R33C mutant developed cortical cataracts at E14 and E20 (Fig. 6C). H&E staining images showed enlarged extracellular spaces (Fig. 6D, black arrowheads) and abnormal nuclei distribution (Fig. 6D, white arrowheads). The disorganization of lens fiber structures became even more apparent in E20 lenses expressing the R33C mutant. These lenses exhibited empty hollow spaces (Fig. 6E, black arrowheads) and greatly increased spaces between lens epithelia and underneath lens fibers (Fig. 6E, asterisks) as well as enlarged lens fibers. This data shows that, in chick lenses, exogenous expression of the human AQP0 mutant that causes congenital cataracts leads to disruption of lens morphology, lens fiber structure and organization, and nuclei disruption, although there was no obvious lens opacity developed during the embryonic development period.

### Mutation of human αA-crystallin (CRYAA) cause reflection changes but not cataract formation

**Fig. 7:**
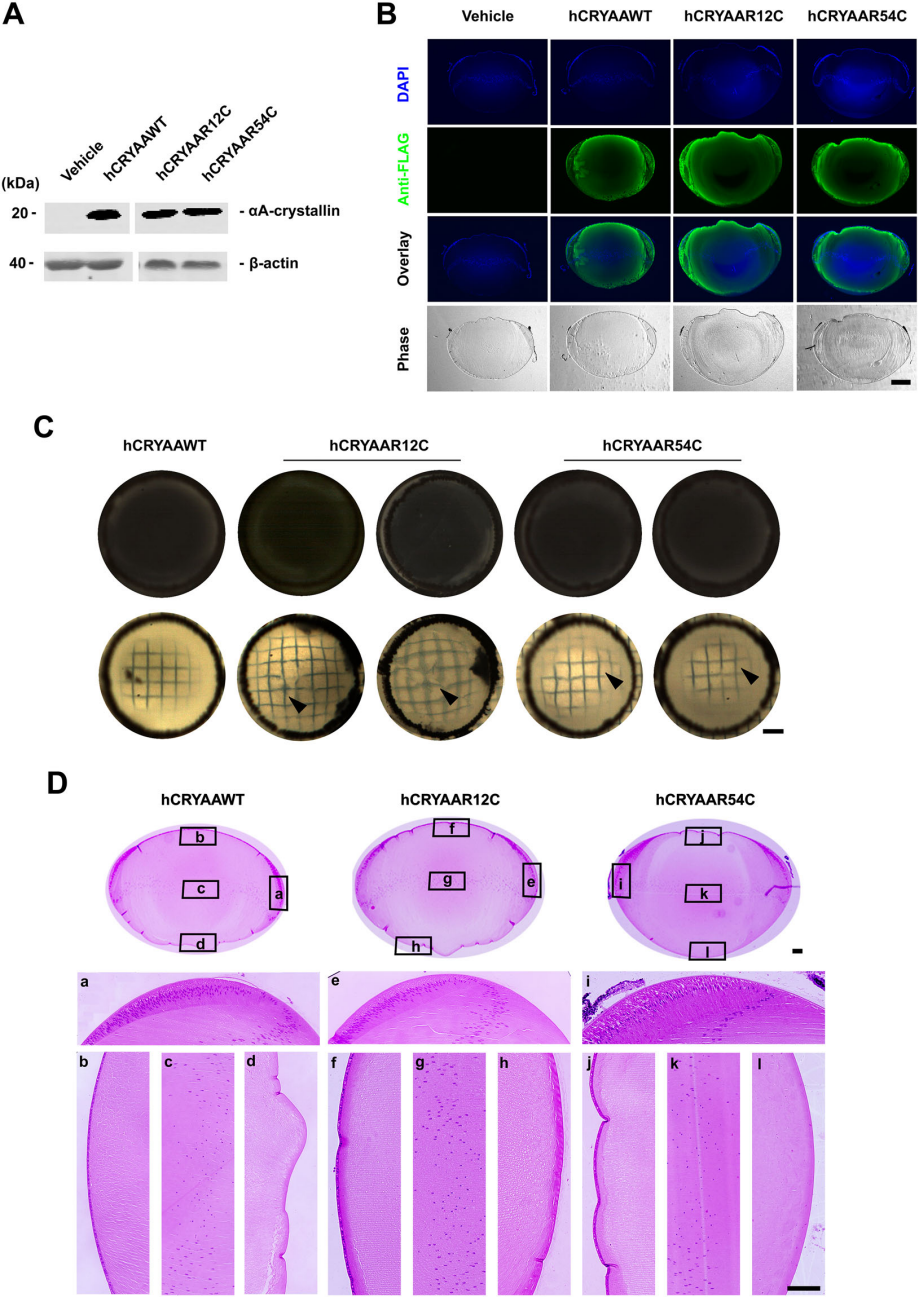
Human crystalline mutations alter the light transmission of the lens. Embryonic chick lenses were injected with recombinant RCAS(A) expressing WT human αA-crystalline, hCRYAA, and two mutations, hCRYAAR12C, and hCRYAAR64C. Lenses were then isolated at E14 and E20. **A** Lens lysates were prepared and immunoblotted with anti-FLAG or anti-β-actin antibody. Scale bar: 400 µm. **B** Paraffin sections of E14 chick lenses were prepared and immunolabeled with anti-FLAG and counterstained with DAPI. Scale bar: 400 µm. **C** Dissected whole E20 chick lenses expressing exogenous WT hCRYAA or one of two mutations were imaged with a dissecting microscope with a black (upper panel) or grid background. **D** The midsagittal paraffin sections of various E20 chick lenses were stained by H&E. Images were captured at a lower resolution for the entire lens and higher resolution for various regions of the lens. Scale bar: 50 µm.

Crystallins (α, β, and γ) are the most abundant cytosolic proteins in the lens and play key roles in lens transparency and light transmission. Crystallin mutations are the leading cause of human congenital cataracts^20^. In addition to being key for lens clarity, α-crystallin is also a chaperone molecule and maintains the membrane structure of lens epithelial and fiber cells^21,22^. Here we exogenously expressed two αA-crystallin (CRYAA) mutants R12C and R54C in the embryonic chick lens. Western blot and immunofluorescence showed comparable expression of WT αA-crystallin and the two mutants in the embryonic lens (Fig. 7A, B and Supplementary Fig. 3). However, no apparent lens opacities were formed in lenses injected with the two αA-crystallin mutants R12C and R54C at E20. Optical images with grids were used to test light transmission. E20 lenses expressing the R12C or R54C mutant exhibited distorted grid lines, while straight lines were shown in lenses expressing WT αA-crystallin (Fig. 7B). H&E staining images showed expression of two mutants, R12C and R54C had no effect on lens structure as compared to WT control (Fig. 7C).

**Fig. 8:**
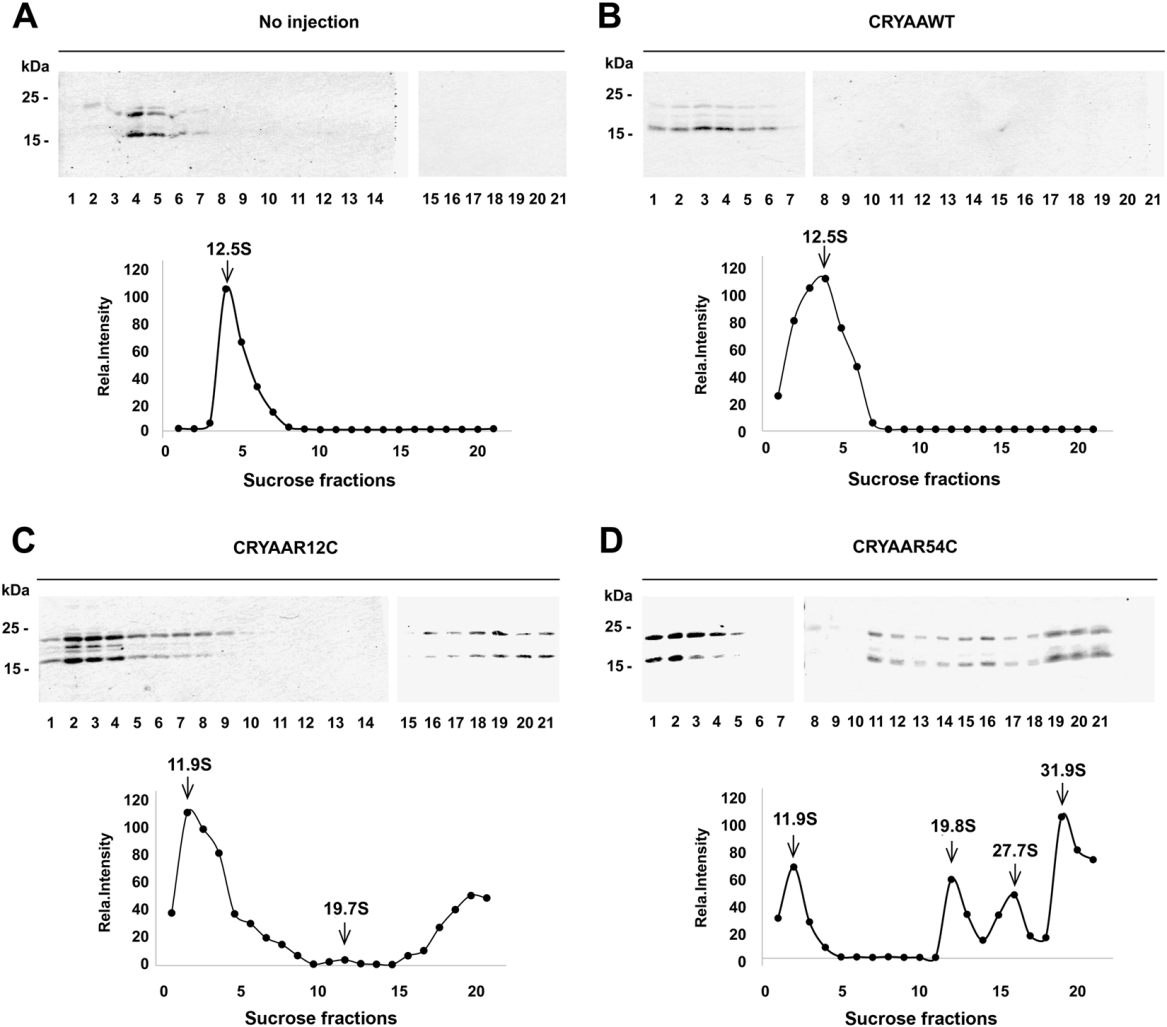
Protein aggregation results from human crystalline mutations exogenously expressed in the embryonic chick lens. Embryonic chick lenses without injection (**A**) or injected with recombinant RCAS(A) expressing WT human αA-crystalline (hCRYAA) (**B**), or one of two mutations, hCRYAAR12C (**C**), and hCRYAAR54C (**D**). Lysate of E20 lenses was prepared and fractionated on a linear gradient of 4–20% sucrose. Each fraction was subjected to SDS/PAGE and western-blotted with an anti-αA-crystallin antibody (upper panels). The sedimentation coefficients were calculated and the band intensity of αA-crystalline in each gradient fraction on western blots was quantified by Image J. and normalized as a percentage of the fraction containing the highest level of hCRYAA or mutations, respectively (bottom panels).

Since αA-crystallin (CRYAA) mutations are reported to cause protein aggregation in congenital cataracts^3,23^, we investigated the extent of protein aggregation in the E20 chick lens using sucrose gradient sedimentation analysis. αA-crystallin in E20 embryonic chick lenses concentrated in a major protein peak with sedimentation coefficient at 12.5S (Fig. 8A and Supplementary Fig. 4, upper panel). Similar to endogenous αA-crystallin, the major peak for exogenous expressed WT was also at 12.5S (Fig. 8B and Supplementary Fig. 4, upper panel). However, the peak was broader probably due to the co-expression of endogenous αA-crystallin. In addition to a peak at 11.9S, there were two additional peaks for R12C mutant, one at 19.7S and the other in the last fraction of the sucrose gradient, these were large aggregates that could not be separated by the gradient (Fig. 8C, Supplementary Fig. 4, bottom panel). Besides the peak shown in WT αA-crystallin, the R54C mutant resulted in multiple forms of protein aggregation peaks at 19.8S, 27.7S, 31.9S, and large aggregates in the last fraction (Fig. 8D and Supplementary Fig. 4, bottom panel). This study shows that like in human congenital cataracts, mutations can lead to the formation of crystalline aggregates in embryonic chick lenses.

## Discussion

With the advancement of human genetics, many causative gene (about 1433) mutations for human congenital cataracts have been identified in the past few decades (June 2020, http://cat-map.wustl.edu/). But very few of the mutated genes have been studied to explore their underlying mechanisms. To have a better understanding of how a specific mutation causes cataracts, a reliable, time and cost-effective animal model is necessary. Moreover, thus far except for surgery intervention, there are no FDA-approved therapeutic drugs available for treating cataracts. Therefore, there is an unmet medical need to develop a non-surgical procedure to treat cataracts, the leading cause of blindness in the world^24^. The embryonic chick model we report here will provide an efficient platform to investigate mechanisms underlying disease development as well as test various potential drug candidates in the treatment of cataracts. In this study, we showed that exogenously expressing known congenital cataract-causing mutants of Cx50, AQP0, and αA-crystalline resulted in the development of nuclear cataracts accompanied by impaired light transmission, disrupted lens structures, and nuclear distribution, and increased protein aggregation. These changes closely recapitulated characteristics of human congenital cataracts. This study demonstrates the utility of this chick embryonic system as a potent in vivo model for cataract research.

In this study, we focused on the exogenous expression of three mutated proteins in embryonic chick lens to test the feasibility of this in situ model system: Cx50, a membrane protein that forms gap junction channels, human AQP0, the most abundant membrane protein in forming water channels, and human αA-crystallin, a soluble cytosolic protein critical for light transmission and lens transparency. Mutations of these three lens proteins and their family members have been identified in the majority of human congenital cataracts^3,25,26^. The research in our laboratory is among the first to use RCAS(A), a replication-competent avian retrovirus, to express exogenous lens proteins in the embryonic chick lens in situ^15,27^. We have previously reported that alkaline phosphatase delivered by recombinant retrovirus is detected in both lenses epithelial and crystalline fiber cells^15^. Consistent with our previous report, we did not observe any exogenous Cx50 expression in lens epithelial cells, while our previous result shows the expression of exogenous Cx43 in the epithelium, but not lens fiber cells^15^. The specific localization pattern of exogenous proteins might be partially explained by unique translational and post-translational mechanisms in lens cells, which have been reported previously^28,29^. Mutants, like what we have shown for WT Cx50^14,15^, were expressed in the majority of lens fiber cells 48 h after retroviral infection. Injection of either retroviral vector or WT lens proteins has minimal effect on lens structure and development as well as lens transparency. However, exogenous expression of site mutants of three lens proteins, which have been identified to be associated with human congenital cataracts in a dominant-negative manner, results in lens opacities, morphological, and structural alterations, and protein aggregation.

The Cx50 mutation E48K was identified in a family of Pakistani origin with congenital “zonular nuclear” cataracts^16^. Using embryonic chick lens and retroviral injection, we recapitulated the formation of nuclear cataracts as a consequence of E48K mutant expression as well as altered lens morphology. Moreover, we showed the degree of lens opacities caused by the mutation is varied ranging from mild to severe lens cloudiness. The heterogeneity in cataracts has been reported in human congenital cataracts with identical mutation among family members and even the counter lateral lens within the same patient. We initially suspected that this difference could be caused by varying levels of the exogenous mutated proteins expressed in the lens. However, we did not detect any major difference in lens samples associated with mild and severe cataracts. Instead, we found that the localization of the mutant in a particular lens region is tightly correlated with the severity of lens opacities. We observed that the abundance of the mutant expressed in the anterior lens region is directly correlated with the severity of lens opacity. Cx50 has a predominant role in gap junction-mediated cell–cell communication in cortical fibers, while Cx46 gap junctions play a major role in nuclear fibers^30,31^. The E48K mutation inhibited gap junction coupling in a dominant-negative manner^32^ Therefore, due to the importance of Cx50 gap junction in outer cortical fibers, expression of dominant-negative mutant Cx50E48K in this region is expected to have a severe effect compared to that of lens nuclear fibers. In addition, elevated oxidative stress indicated by 4-HNE levels is also associated with lenses expressing Cx50E48K. Oxidative stress is the major cause of nuclear cataracts^18^. Moreover, oxidative stress has been suggested to have an important role in the onset of congenital cataracts^33^. The elevated oxidative stress in lens expressing Cx50E48K might be the main cause of lens opacity.

Similar to the Cx50 mutation, we show that a human congenital causative mutant of human AQP0 also developed cataract formation associated with disruption of lens fiber structures in this embryonic chick lens model. We exogenously expressed the R33C site mutant of AQP0. This mutant has a dominant-negative effect on cell–cell adhesion and cataract formation but has no effect on trafficking and membrane localization of AQP0^34,35^. Here, we show that exogenous expression of R33C disrupted lens structure with the formation of large extracellular spaces and abnormal distribution of fiber cell nuclei in the chick lens. Additionally, lens epithelial cells were separated from underneath fiber cells with an enlarged space in between. These phenomena likely attribute to the impaired cell–cell adhesion function of AQP0^36^. Similar to in vitro experiments^34,36–38^, mutant, like WT AQP0, in chick lens showed plasma membrane localization, consistent with normal trafficking of the mutant. However, puncture staining of the AQP0 mutant shown by immunofluorescence indicated abnormal protein aggregation, such as abnormal association with other proteins (e.g., αA-crystallin) as previously reported^39^.

In contrast to the mutations of Cx50 and AQP0, exogenous expression of the αA-crystallin (CRYAA) mutants, R12C or R54C in embryonic chick lenses only impaired normal light transmission without apparent development of lens opacities. The R12C mutation in human CRYAA has been reported to exhibit heterogeneities in cataract and other eye phenotypes, such as nuclear and laminar cataracts, and some families present with microcorneas^40,41^. In addition, Hansen et al. reported that the R12C mutation was associated with congenital cataracts, showing posterior polar opacity progressing to dense nuclear and laminar cataracts^42^. This partially explains why cataracts were not observed in embryonic chicks with R12C expression. The R54C mutation in the human CRYAA gene has been linked to nuclear cataracts and microcorneas^43^. The R54C mutation is also reported to exhibit a high level of heterogeneity in cataract formation as shown in members of two families with nuclear cataracts with varied genetic penetrance^43,44^. Additionally, the development of cataracts by the R54C mutation in homozygous mice occurs early at postnatal 3 weeks and is associated with structural disruptions of epithelial cells and degenerated fiber cells^45^. It is plausible that the embryonic time frame is not sufficient to develop lens opacities for these two crystallin mutants. However, based on this study, we observed that morphological and structural changes occurred much earlier than visible lens cloudiness. Moreover, these lenses show deficits in light transmission, which indicates an early stage of cataract formation^46^. According to clinical studies, patients under 6 years of age account for about 70% of total cases of congenital cataracts^47^. Due to the early lens differentiation, organelle degeneration and denucleation of lens fibers, the embryonic chick lens at the late developmental stage exhibits characteristics of the lens as seen in the postnatal human lens. This infers that the majority of cataracts or at least certain lens defects in chick may appear at embryonic stages. Furthermore, even the same congenital cataract mutations could result in different onset among patients^26^. Another possibility is that endogenous crystallin may compensate for the alterations caused by exogenous crystallin mutants as co-expression of αB-crystallin inhibited protein aggregations caused by an αA-crystallin mutation^48^. Nevertheless, we observed the distortion of the lens refractive property. Moreover, sucrose gradient sedimentation analysis unveiled mutant αA-crystallin protein aggregation commonly shown in cataracts associated with crystallin mutants. The sedimentation coefficient of αA-crystallin in non-injected and injected lens samples is around 12.5S, which is similar to the reported value for recombinant αA-crystallin^49^. This data confirmed that injection of WT crystallin had no effect on the oligomeric state of αA-crystallin. Contrary to WT, R12C or R54 mutations resulted in the aggregation of crystallin molecules as reflected by the increased sedimentation coefficient values by sucrose gradient sedimentation. The extent of protein aggregation induced by the R12C and R54C mutations was relatively moderate and is comparable to the sedimentation coefficients seen in elderly patients without apparent cataracts^49^. This may partially explain the lack of visible cataracts observed in our system with these mutants. These studies reflect that increased accumulation of αA-crystallin aggregation and/or other post-translational modifications ultimately leads to cataracts.

It is reported that 85% of congenital cataracts are inherited as an autosomal-dominant trait, suggesting that the majority of cataract mutations likely function in a dominant-negative manner^26^. Therefore, exogenous expression of dominant-negative mutations in our model will likely mimic the majority of congenital cataracts. Our model may not directly address the action of non-dominant-negative trait mutations. To address non-dominant mutations interference RNA (RNAi) may be used via recombinant retroviral injections to specifically knockdown a gene to study its function, a method that has been previously reported^50^. In those studies, recombinant retrovirus was reported to deliver interference RNA (RNAi) targeting visinin expression in the retina, although no phenotype was observed in that study^50^. In addition to the exogenous expression of dominant-negative mutants, the embryonic lens has been used to overexpress other proteins. For example, overexpression of bcl-2 leads to delayed cell denucleation without cataract formation^51^. In this study, only E8 lenses were observed. Another study reports that delivery of a small hairpin RNA into the cranial region knocked down C-X-C motif ligand 14 (CXCL14) expression, resulting in neuropatterning defects and neovascularization of the cornea^52^. The phenotypes of overexpressing WT proteins could be overshadowed by the co-expression of endogenous counterparts in the embryonic chick system. Knocking down genes with RNA interference in this system is an alternative to studying specific gene functions, although the majority of human genetic diseases are caused by gene mutations and not necessarily loss of the protein. Given that almost all genes that cause human congenital cataracts present as an autosomal-dominant trait, the embryonic chick lens provides an ideal model system to investigate underlying mechanisms and to develop treatment modalities.

Chicken eggs have been well established as a model for drug screening and toxicity testing^53–57^. In this model, the treatment could be performed readily with the administration of reagents to the chorioallantoic membrane, and the opening of the eggshell can be easily sealed afterward, allowing for repeated drug applications. Treatment for cataracts has been previously reported in embryonic chicks at embryonic day 15 by a single injection in a steroid-induced cataract model^58,59^. In our study, cataract formation by overexpression of dominant-negative mutants appears as early as E14 and continues the progression of lens opacity to E21 before hatching and this time frame permits us to test the efficiency of the therapeutic candidates. Taken together, this model could be used as a model for drug screening and discovery for cataracts in a time and cost-effective manner.

In summary, based on the studies of mutations of three representative lens proteins, our study suggests that the embryonic chick lens along with the effective retroviral delivery approach used here, provide an effective and potent in vivo system to investigate various mutations that cause congenital cataracts. Moreover, the experimental model described here will allow studying structure-function and molecular mechanisms of lens proteins in normal lens physiology and development. Additionally, this model can potentially be used for drug screening and identifying therapeutic candidates for treating human cataracts. Furthermore, this system provides an in situ microenvironment, which can be used to study protein aggregation and modifications. In our study, the puncta formation in the AQP0 mutation and protein aggregation in the CRYAA mutation indicates that this model could be used to study protein aggregation in situ. In addition to the research in lens cataract formation, this model could potentially be developed as an in vivo platform for studying other non-lens proteins. Post-translational protein modifications are a leading cause of human diseases, such as cataracts, Alzheimer’s disease, and prion disease^60^. The data presented in this study support the feasibility of using this experimental model to study the causative mechanism of cataracts and other diseases and offers a unique platform for potential therapeutic development. Therefore, the potential application of this model may be broad beyond lens proteins and therefore warrants further research and development.

## Methods

### Materials

Fertilized white leghorn chicken eggs were obtained from the Department of Agriculture & Poultry Science, Texas A&M University (College Station, TX) and incubated in a humidified chicken egg incubator at 37 °C. Rabbit anti-chick Cx50 and rabbit anti-chick AQP0 polyclonal antibodies were generated and affinity purified^61^. Rabbit anti-FLAG tag antibody (Cat#600-401-383) was obtained from Rockland Immunochemicals (Pottstown, PA). Mice anti-FLAG monoclonal antibody (F1804) was purchased from Sigma (St Louis, MO). Anti-CRYAA (ab5595) antibody was obtained from Abcam (Cambridge, UK). Paraformaldehyde (PFA, 16%) was purchased from Electron Microscope Science (Fort Washington, PA). Dulbecco’s Modified Eagle Medium (DMEM), 0.25% Trypsin-EDTA solution and penicillin/streptomycin were purchased from Invitrogen (Carlsbad, CA, USA). Fetal bovine serum (FBS) was obtained from Hyclone Laboratories (Logan, UT, USA). The nitrocellulose membrane was obtained from Bio-Rad (Hercules, CA). DNA plasmids contain human CRYAA (hCRYAA) was obtained from Addgene (Watertown, Massachusetts). DNA plasmids contain human AQP0(hAQP0) insert with a fluorescent tag at the C-terminal end was generated by Dr. Kulandaiappan Varadaraj as described before^34^. All other chemicals were obtained from either Sigma or Fisher Scientific (Pittsburgh, PA).

### Preparation of high-titer recombinant retroviruses

Chick embryonic fibroblast (CEF) cells were prepared^14^, and tested for possible mycoplasma contamination. Recombinant retroviral DNA constructs and high-titer recombinant retroviruses containing chick wild type (WT) Cx50 or mutant Cx50E48K were prepared based on our published protocol^32,62^. Briefly, 1.5 µg of DNA constructs containing connexins were transfected into CEF cells cultured in 60 mm plates at 50–70% confluency using lipofectamine according to the manufacturer’s instructions (ThermoFisher Scientific). CEF cells transfected with these DNA constructs were cultured, and conditioned media was collected and concentrated to generate high titer recombinant retrovirus. To examine the expression of connexins, crude membrane extracts of transfected CEF cells were prepared and immunoblotted with rabbit anti-FLAG antibody (1:1000 dilution).

To generate recombinant retroviral AQP0 mutant-RCAS(A) constructs, plasmids containing WT human aquaporin 0 (hAQP0) or site mutant hAQP0R33C (the mutation of Arg residue at position 33 to Cys), and an adaptor plasmid Cla12NCO, were digested with *Xba*I. The DNA fragments were isolated through agarose gel isolation and ligated to generate a Cla12NCO-AQP0 DNA construct. The Cla12NCO construct was digested with *Cla*I, and released AQP0 fragments were subcloned into a ClaI-linearized RCAS(A) retroviral vector.

For the construction of chimeric retroviral constructs, WT human CRYAA (hCRYAAWT), an hCRYAAWT fragment containing carboxyl-termini FLAG tag and restriction enzyme site was generated by PCR using the following primer pairs: sense, 5′-ACTGCCATGGATGTGACCATCCAGCA-3′; antisense, 5′-CAGTAAGCCTTACTTGTCATCGTCGTCCTTGTAGTCGGACGAGGGAGCCGAGGTG-3′. PCR fragments were ligated into a Cla12NCO vector by digestion with *NCO*I and *Hin*dIII, and then isolated through agarose gel isolation to generate Cla12NCO-hCRYAAWT constructs. Using the QuickChange site-directed mutagenesis kit (Stratagene, La Jolla, CA), the site mutation of Arg at position 12 to Cys (R12C) or Arg at position 54 to Cys (R54C) was introduced with the following primer pairs: sense, 5′-CCCTGGTTCAAGTGCACCCTGGGGC-3′; antisense, 5′-GCCCCAGGGTGCACTTGAACCAGGG-3′; sense, 5′-CCAGTCCCTCTTCTGCACCGTGCTGGA-3′; antisense, 5′-TCCAGCACGGTGCAGAAGAGGGACTGG-3′, respectively. Finally, the Cla12NCO-CRYAA constructs were isolated after digestion with ClaI and subcloned into a ClaI-linearized RCAS(A) retroviral vector. The constructs with the correct orientation of inserted fragments were confirmed by digestion with ClaI and SalI restriction enzymes. All DNA PCR primers were synthesized at Integrated DNA Technologies (Coralville, IA). All the constructs generated were sequenced at GENEWIZ (South Plainfield, NJ) to ensure the correctness of the sequences. High-titer recombinant retroviruses were generated through transfection of these constructs into CEF cells^14,15^.

### Chick lens microinjection

High-titer recombinant retroviruses containing various WT and mutant lens protein cDNAs were prepared as described above. Microinjection of retroviruses into the lens of chicken embryos was based on our published procedure^15^. Briefly, chicken eggs were opened under the sterile conditions at the developmental stage 18 (~65–68 h of incubation). The amniotic membrane was removed to provide unimpeded access to the developing eye. About 20 nl of concentrated viral stock was directly microinjected into the lumen of the right lens vesicle using a glass pipette (tip opening diameter ~10 μm). Retroviral stocks were colored with Fast Green to visualize the filling of the lens vesicle lumen. The left lens was kept intact to serve as a contralateral control. After injection, the eggshell opening was sealed with tape and the embryo was returned to 37 °C for further incubation. The injected lenses were dissected on an embryonic day (E) 4, 5, 7, 11, 14, and 18 and imaged with the anterior side facing up by using a standard dissection microscope.

### Lens tissue paraffin sections and histochemistry

All animals were housed and studied in accordance with NIH Animal Care and Use Committees (ACUC) guidelines, and the animal protocols were approved by the University of Texas Health Science Center at San Antonio (UTHSCSA) Institutional Animal Care and Use Committee (IACUC). Eyeballs or lenses were isolated, fixed in 2% paraformaldehyde (PFA), and incubated overnight at 4 °C. Lenses were then dehydrated with ethanol and xylene, embedded in paraffin, and sectioned 3 µm in thickness. The tissue sections were mounted to glass slides and stained with hematoxylin and eosin (H&E). For 4-hydroxynonenal (4-HNE) staining, paraffin slides were antigen retrieved using sodium citrate buffer (pH 6.0) at 65 °C for 2 h. Then the ABC (avidin-biotin-peroxidase complex) Immunostaining Assay kit was used^63^. Images were recorded at ×20 magnification using a Keyence BZ-X710 microscope (Osaka, Japan).

### Lens frozen tissue sections and immunofluorescence

The dissected embryo lenses were fixed in 2% PFA and incubated overnight at 4 °C, dehydrated with sucrose, and embedded in Tissue-Tek compound. Sagittal sections (12 mm) were collected and stored at −20 °C. For lenses injected with hAQP0WT or hAQPR33C, whole lens images were captured with a Keyence BZ-X710 microscope before fixation. For immunolabeling of exogenous proteins, lens sections were first incubated in phosphate-buffered saline (PBS) for 5 min, then in blocking solution (2% normal goat serum, 2% fish skin gelatin, 0.25% Triton X-100 and 1% bovine serum albumin (BSA) in PBS) for 1 h, and finally in blocking solution containing monoclonal anti-FLAG antibody (1:1000 dilution) overnight at 4 °C. Sections were washed three times, 5 min each time in PBS, and then incubated with fluorescein-conjugated donkey anti-mouse IgG against anti-FLAG (1:500 dilutions in blocking solution) for 2 h at room temperature. After three washes in PBS for 5 min each, a drop of mounting medium was added before being covered by a glass coverslip. The images of specimens were taken using a Keyence BZ-X710 microscope. Imaging acquisition conditions were kept constant for all samples.

### Sucrose gradient sedimentation analysis

E20 day lenses injected with or without WT and mutant CRYAA were homogenized in ice-cold lysis buffer containing 1 mM PMSF, 1 mM NaVO4, and 0.1 mM leupeptin. Lens lysates were fractionated on a linear gradient of 4–20% sucrose (wt/vol at20 °C) with a total volume of 5 ml in the presence of 10 mM Hepes, pH 7.2, and 0.5% octylpolyoxyethylene (8-POE). Centrifugation was performed in a Beckman SW41Ti rotor at 33,600 rpm (139,404 × *g*) for 16 h at 4 °C, and then 500 µl fractions were collected. Western blots were performed by probing with rabbit anti-CRYAA (1:5000 dilution). Primary antibodies were detected with horseradish-peroxidase-conjugated goat anti-rabbit IgG (1:5000 dilution) using a chemiluminescence reagent kit (ECL). The intensity of the bands on western blots was quantified by Licor (Lincoln, NE). The sedimentation coefficient of crystallin was calculated according to published methods^64^. Briefly, two formulas were applied; the first formula *Z*_0_ = (*Z*_1_*r*_2_ − *Z*_2_*r*_1_)/(*r*_2_ − *r*_1_) was used to calculate the solute concentration corresponding to extrapolation of a linear gradient distribution to a zero radius (*Z*_0_). In this formula, *Z*_1_ is the minimum percent of sucrose gradient, which is 4% in our study, and *Z*_2_ is the maximum percent of sucrose gradient which is 20%. *r*_1_ and *r*_2_ are the minimum and maximum radial distance from the centrifugal axis (cm), respectively. Then the *Z* value and tables presented in McEven et al.^64^ were used to obtain the time integral *I*_1_ for sucrose at the meniscus of the gradient and *I*_2_ at the separated zone for the particle. Finally, the sedimentation coefficient was calculated from formula *S* = (*I*_2_ − *I*_1_)/ω^2^*t*, where ω is the speed of the rotor (2πs^−1^) and *t* is the centrifugation time (s).

### Statistical analysis and reproducibility

Data were analyzed with one-way ANOVA and Turkey’s multiple comparison test along with GraphPad Prism (GraphPad Software, La Jolla, CA). Data are presented as the mean ± SEM of multiple measurements. Asterisks represent the degree of significance in comparison with controls (*P < 0.05; **P < 0.01; ***P < 0.001). Some of the data presented were normalized to controls for comparison, and the variables were analyzed as described above. At least three independent experiments (*n* ≥ 3) were performed.

## Supplementary information

Supplementary Information

Supplementary Data 1

Peer Review File

## Data Availability

The source data for uncropped western blots and source data underlying all graphs and charts for main figures presented in this paper are available as Supplementary Data 1. Any remaining information can be obtained from the corresponding author upon reasonable request.
