# Supplementary material for: Development of a potent embryonic chick lens model for studying congenital cataracts in vivo
Source: Commun Biol. 2021 Mar 11;4:325. doi: 10.1038/s42003-021-01849-0 (PMC7952907; doi:10.1038/s42003-021-01849-0)
Supplement: Supplementary file 1 — Supplementary Information [file 42003_2021_1849_MOESM1_ESM.pdf]

Supplemental Figures

Development of a Potent Embryonic Chick Lens Model for Studying Congenital Cataracts *in vivo*

[1]Zhen Li, [1]Sumin Gu, [1]Yumeng Quan, [2]Kulandaiappan Varadaraj, and [1]*Jean X. Jiang

[1]Department of Biochemistry and Structural Biology, University of Texas Health Science Center, San Antonio, Texas 78229, USA.
[2]Department of Physiology and Biophysics, Stony Brook University, Stony Brook, New York 11794, USA

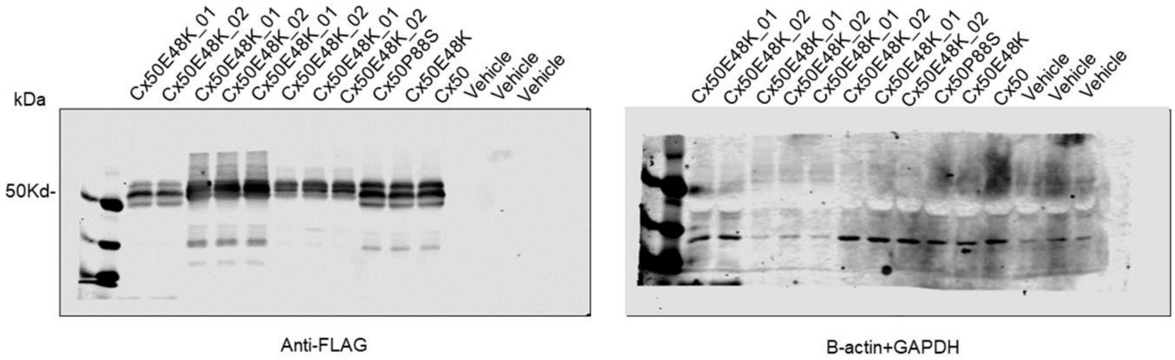

Supplementary Figure 1. Raw western blots data for Figure 1. Chick embryonic lenses after retroviral infection were collected and crude membrane extracts were prepared. The expression of WT Cx50 and Cx50E48K mutant was detected by western blotting using anti-FLAG tag antibody. Lysates of E14 chick lenses injected with retrovirus containing Cx50P88S or Cx50E48K (Cx50E48K clones 01 and 02, representing lenses with mild and severe cataracts, respectively) as labeled were loaded on SDS-PAGE and immunoblotted.

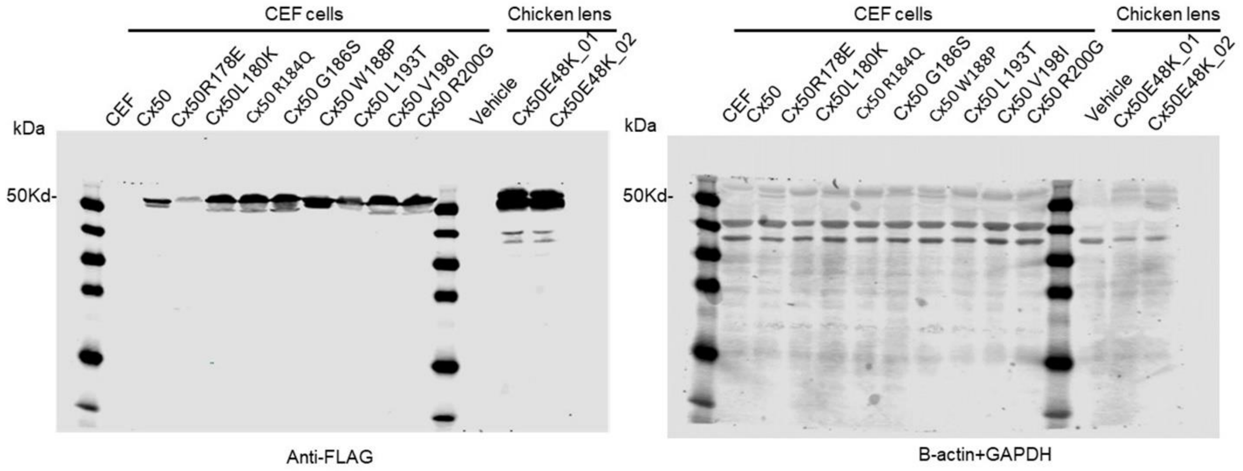

Supplementary Figure 2. Raw western blots data for Figure 5. Lysates of E14 chick lenses injected with retrovirus containing vector or Cx50E48K (Cx50E48K_clones 01 and 02, representing lenses with mild and severe cataract, respectively) as labeled were immunoblotted with anti-FLAG or anti-β-actin antibody. Lysates of CEF cells with or without infection were loaded on SDS-PAGE and immunoblotted.

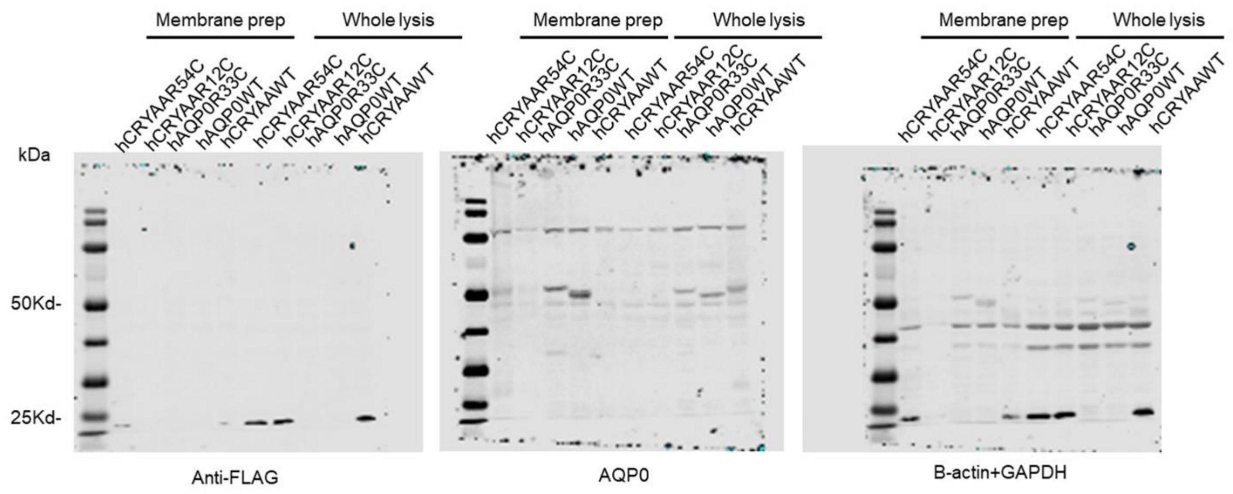

Supplementary Figure 3. Raw western blots data for Figure 6 and Figure 7. Lysates of E14 chick lenses were prepared, loaded on SDS-PAGE and immunoblotted with anti-AQP0, anti-FLAG or anti-β-actin antibody.

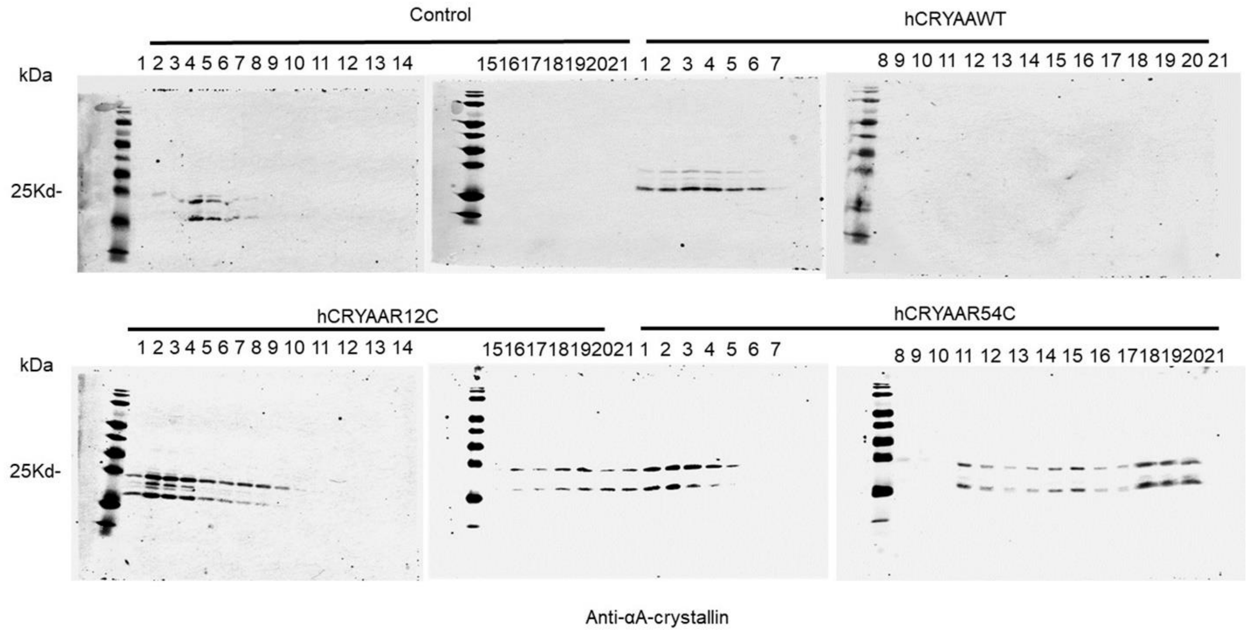

Supplementary Figure 4. Raw western blots data for Figure 8. Lysates of E20 lenses were prepared and fractionated on a linear gradient of 4-20% sucrose. Each fraction was subjected to SDS/PAGE and immunoblotted with anti-αA-crystallin antibody.
