# Supplementary material for: Development of a potent embryonic chick lens model for studying congenital cataracts in vivo
Source: Commun Biol. 2021 Mar 11;4:325. doi: 10.1038/s42003-021-01849-0 (PMC7952907; doi:10.1038/s42003-021-01849-0)
Supplement: Supplementary file 3 — Peer Review File [file 42003_2021_1849_MOESM3_ESM.pdf]

Reviewers' comments:

Reviewer #1 (Remarks to the Author):

The authors present an intriguing and novel model to study the onset of congenital cataracts. They utilize a unique window in chick lens development combined with RCAS virus for transgene expression of human proteins associated with cataract formation. They convincingly show that their model exhibits cataractous lenses that mimic several properties congenital cataracts in humans. This model could serve as a useful platform to study the formation of cataracts and would have positive impact on the field. ¬¬

Below are my major concerns and minor comments:

Major concerns:

The authors argue that this new model for congenital cataracts circumvents limitations of current models, which include expensive and time-consuming mouse models. They then proceed to claim that pharmacological drug screening in cheaper in vivo models could efficiently identify drug candidates for treatment. While the model presented here is a new and exciting way to introduce cataracts in embryos and study mechanisms of cataract formation, it is not clear how this technology could be used to screen for drugs that might be useful therapeutics. If the arguments for this study stated in the introduction are to be supported, the authors should discuss how this system permits more efficient or cost-effective drug discovery in the discussion of this manuscript.

The authors state that some of the CRYAA mutations did not result in embryonic cataracts in the chick lens, but may lead to cataracts after hatching. How many congenital cataract mutations result in embryonic cataracts relative to postnatal onset of cataracts? Hatching chickens, and the required chicken husbandry, would become costly. This directly impacts the original objective of creating a cost-effective in vivo model to identify drug candidates to treat this disease. The authors should discuss that their model, as presented, is limited to studying and screening treatments for fetal congenital cataracts.

This model is quite effective in generating cataractous lenses by overexpressing proteins containing mutations associated with congenital cataracts. But how well does this model recapitulate diseases? It would be useful if the authors clearly drew comparisons between all phenotypes observed in their model (for example, lens fiber disorganization, the correlation of altered Cx50E48K protein distribution and cataract severity). Moreover, the phenotypes shown in this manuscript are all caused by the overexpression of proteins with dominant negative mutations. Out of the ~1440 mutations for human congenital cataracts (referenced in this manuscript), how many are dominant negative? Addressing this is important to support the usefulness of this system to model human disease.

The authors claim that WT-AQP0 chick lenses 'showed plasma membrane localization consistent with normal trafficking of the protein' while the R33C-AQP0 mutation shows 'punctate staining…indicating abnormal protein aggregation'. The data presented in Fig. 6B does not convincingly support of these claims. These tissues must be imaged at much higher magnification and aggregated protein puncta must be quantified (puncta/area or volume).

Minor comments:

The authors routinely refer to the constructs containing cataract-causing mutations as mutants. They

should refer to the mutation and not say mutant.

Figure 1: The authors inject the lens vesicle with the viral constructs, which would suggest that both the lens epithelium and crystalline cells would be equally infected and show transgene/protein expression. However, the data shows that only the crystalline cells express the recombinant proteins. Also, since most of the proteins in this study are associated with crystalline cells, what would happen if they are ectopically expressed in the epithelial cells?

Figure 3B: Please state how the opacity is normalized on the y-axis. It is not clear what the values on the y-axis represent. If normalized to control, then the value should be 1? Is this a measurement of transparency or opacity?

Opacities in the Crystallin mutants and AQP mutations are less obvious. Please quantify lens opacity in these lenses as you did for the Cx50 mutations.

Reviewer #2 (Remarks to the Author):

This manuscript describes the development of approaches to induce congenital cataracts in chicken embryos in ovo. Using injection into the lens lumen of ~HH18 chicken embryos of RCAS virus constructs, the authors demonstrate that exogenous expression of the Cx50E48K and AQPOR33C mutations associated with congenital cataract in humans results in cataract formation in chicken embryos. Structural defects in the lens are described. In contrast, ectopic expression of alpha crystallin mutations associated with cataract in humans did not result in cataract in the chicken, at least at the time points analysed. However, light transmission and protein aggregation were altered, which can be forerunners of cataract development. The authors propose that these in vivo models of congenital cataract could be used to study mechanisms of cataract formation and as a tool to screen for therapeutic interventions.

The paper is well written and the data analyses are, in the main, robust. The images are of a very high standard and the figures well laid out and clearly presented. The statistical analyses seem appropriate and sufficient data is presented in the methods section to enable the work to be reproduced (although this will require referring to previously published work).

The main limitation of the study is that it is purely descriptive. The mechanism(s) by which cataracts are induced by the mutant constructs is not established. The extent to which the manuscript advances understanding therefore is quite limited. This said, the description of the model is in itself a potentially useful advance and may be of benefit to others. Overall, given the aim of this manuscript is to disseminate information on a method for generating congenital cataracts in chicken, my feeling is that this paper may be more suitable for a specialist eye journal.

Minor point:

1. The abnormal nuclear organisation that occurs would benefit from some quantification. For example, on P6, lines 7-8 it is stated that “at E4 it appeared to have more nuclei”. Quantification at different ages of the total number of nuclei, their abnormal location and % of nuclei that are aberrantly positioned would allow more robust conclusions to be drawn.

Responses to review

Referee #1

The authors present an intriguing and novel model to study the onset of congenital cataracts. They utilize a unique window in chick lens development combined with RCAS virus for transgene expression of human proteins associated with cataract formation. They convincingly show that their model exhibits cataractous lenses that mimic several properties congenital cataracts in humans. This model could serve as a useful platform to study the formation of cataracts and would have positive impact on the field. Below are my major concerns and minor comments

Response: We greatly appreciate the positive comments, thorough evaluation of our work, and constructive comments and suggestions.

Major concerns:

The authors argue that this new model for congenital cataracts circumvents limitations of current models, which include expensive and time-consuming mouse models. They then proceed to claim that pharmacological drug screening in cheaper in vivo models could efficiently identify drug candidates for treatment. While the model presented here is a new and exciting way to introduce cataracts in embryos and study mechanisms of cataract formation, it is not clear how this technology could be used to screen for drugs that might be useful therapeutics. If the arguments for this study stated in the introduction are to be supported, the authors should discuss how this system permits more efficient or cost-effective drug discovery in the discussion of this manuscript.

Response: We appreciate the reviewer's comment on this issue. So far, we haven't had any experimental evidence to support this claim. We have revised this part by including additional discussions regarding how this model may help the process of drug screening and discovery, especially in identifying drug candidates in treating cataracts caused by specific gene mutations. (Page 21, lines 15-23).

The authors state that some of the CRYAA mutations did not result in embryonic cataracts in the chick lens, but may lead to cataracts after hatching. How many congenital cataract mutations result in embryonic cataracts relative to postnatal onset of cataracts? Hatching chickens, and the required chicken husbandry, would become costly. This directly impacts the original objective of creating a cost-effective in vivo model to identify drug candidates to treat this disease. The authors should discuss that their model, as presented, is limited to studying and screening treatments for fetal congenital cataracts.

Response: As the reviewer pointed out, hatching chicken and chicken husbandry would become costly as some of the mutations did not result in visible embryonic cataracts in the chick lens. However, these lenses still show deficits in light transmission, which indicates an early stage of cataract formation (Hockwin 1994). According to clinical studies, patients under 6 years of age account for about 70% of total cases of congenital cataracts (Lin, Yang et al. 2014) while the lifespan of *Gallus gallus domesticus* is only about 6-7 years. This infers that the majority of cataracts or at least certain lens defects in chick may appear at embryonic stages. Moreover, even the same congenital cataract mutations could result in different onset among patients (Shiels and Hejtmancik 2019). We have included the above in the Discussion (Page 19, lines 15-21).

This model is quite effective in generating cataractous lenses by overexpressing proteins containing mutations associated with congenital cataracts. But how well does this model recapitulate diseases? It would be useful if the authors clearly drew comparisons between all phenotypes observed in their model (for example, lens fiber disorganization, the correlation of altered Cx50E48K protein distribution and cataract severity). Moreover, the phenotypes shown in this manuscript are all caused by the overexpression of proteins with dominant negative mutations. Out of the ~1440 mutations for human congenital cataracts (referenced in this manuscript), how many are dominant negative? Addressing this is important to support the usefulness of this system to model human disease.

<u>Response:</u> The reviewer raised a good point about this model. First, most of the studies explore the gene mutations which lead to congenital cataracts, but very few studies have investigated morphological and structural changes, and mechanisms as the result of the mutations. The primary reasons are due to limitations of clinical samples and very few animal models available for cataract mutants. For the mutations we studied, lens expressing Cx50E48K exhibited a similar pattern of “zonular nuclear” cataract as reported in human patients. We have included the description in the Discussion (Page 17, lines 7-10). To date, only *in vitro* studies demonstrate that Cx50E48K inhibited gap junctions in a dominant negative way (Banks, Toloue et al. 2009).Therefore, the model described in this paper will provide a tool to address these gaps. Secondly, it is reported that 85% of congenital cataracts are inherited as an autosomal dominant trait, suggesting that the majority of cataract mutations likely function in a dominant negative manner (Shiels and Hejtmancik 2019). Lastly, if a mutation is not dominant negative, overexpression of the mutant is less feasible. However, though it may not directly address the function of a given mutation, interference RNA (RNAi) can be used via recombinant retrovirus to specifically knock down a gene to study its function as previously reported (Harpavat and Cepko 2006). We have incorporated the above in the Discussion (Page 20, lines 14-21).

The authors claim that WT-AQP0 chick lenses ‘showed plasma membrane localization consistent with normal trafficking of the protein’ while the R33C-AQP0 mutation shows ‘punctate staining...indicating abnormal protein aggregation’. The data presented in Fig. 6B does not convincingly support of these claims. These tissues must be imaged at much higher magnification and aggregated protein puncta must be quantified (puncta/area or volume).

<u>Response:</u> As suggested by the reviewer, we have quantified the aggregation protein puncta and shown the data with tissue images in Figure 6B. The major abnormality is protein aggregation which we have demonstrated by the sucrose gradient experiments. The immunostaining shows the difference between the WT and the mutation, which may be caused by protein aggregation. In the revision, we have quantified lens opacity (Figure 6C) and the increased level of opacity appears to correlate with the number of puncta.

Minor comments:

The authors routinely refer to the constructs containing cataract-causing mutations as mutants. They should refer to the mutation and not say mutant.

Response: We have replaced the term as suggested.

Figure 1: The authors inject the lens vesicle with the viral constructs, which would suggest that both the lens epithelium and crystalline cells would be equally infected and show transgene/protein expression. However, the data shows that only the crystalline cells express the recombinant proteins. Also, since most of the proteins in this study are associated with crystalline cells, what would happen if they are ectopically expressed in the epithelial cells?

Response: As the reviewer pointed, both the lens epithelium and crystalline cells would be equally infected. We have previously reported that alkaline phosphatase delivered by recombinant retrovirus is detected in both lens epithelial and crystalline fiber cells (Jiang and Goodenough 1998). Consistent with our previous report, we did not observe any exogenous Cx50 expression in lens epithelial cells, while our previous results show the expression of exogenous Cx43 in epithelium, but not crystallin cells (Jiang and Goodenough 1998). The specific localization pattern of exogenous proteins might be partially explained by unique translational and posttranslational mechanisms in lens cells, which have been reported previously (Beebe and Piatigorsky 1981, Jiang, Paul et al. 1993). We have included the above in the Discussion (Page 16, lines 16-23).

Figure 3B: Please state how the opacity is normalized on the y-axis. It is not clear what the values on the y-axis represent. If normalized to control, then the value should be 1? Is this a measurement of transparency or opacity?

Response: The result of Figure 3B is the measurement of opacity. The opacity is normalized to the opposite, contralateral eye, and we have included the information in the corresponding figure legend.

Opacities in the Crystallin mutants and AQP mutations are less obvious. Please quantify lens opacity in these lenses as you did for the Cx50 mutations.

Response: Opacities in AQP0 mutation has now been quantified and provided in a new Figure 6C. Since there is no cataract formation in lenses with the crystallin mutation, the quantification was not performed.

References:

Banks, E. A., M. M. Toloue, Q. Shi, Z. J. Zhou, J. Liu, B. J. Nicholson and J. X. Jiang (2009). "Connexin mutation that causes dominant congenital cataracts inhibits gap junctions, but not hemichannels, in a dominant negative manner." J Cell Sci **122**(Pt 3): 378-388.

Beebe, D. C. and J. Piatigorsky (1981). "Translational regulation of delta-crystallin synthesis during lens development in the chicken embryo." Dev Biol **84**(1): 96-101.

Harpavat, S. and C. L. Cepko (2006). "RCAS-RNAi: a loss-of-function method for the developing chick retina." BMC Dev Biol **6**: 2.

Hockwin, O. (1994). "Cataract classification." Doc Ophthalmol **88**(3-4): 263-275.

Jiang, J. X. and D. A. Goodenough (1998). "Retroviral expression of connexins in embryonic chick lens." Invest Ophthalmol Vis Sci **39**(3): 537-543.

Jiang, J. X., D. L. Paul and D. A. Goodenough (1993). "Posttranslational phosphorylation of lens fiber connexin46: a slow occurrence." Invest Ophthalmol Vis Sci **34**(13): 3558-3565.

Lin, H., Y. Yang, J. Chen, X. Zhong, Z. Liu, Z. Lin, W. Chen, L. Luo, B. Qu, X. Zhang, D. Zheng, J. Zhan, H. Wu, Z. Wang, Y. Geng, W. Xiang, W. Chen, Y. Liu and C. S. Group (2014). "Congenital cataract: prevalence and surgery age at Zhongshan Ophthalmic Center (ZOC)." PloS one **9**(7): e101781-e101781.
Shiels, A. and J. F. Hejtmancik (2019). "Biology of Inherited Cataracts and Opportunities for Treatment." Annual review of vision science **5**: 123-149.

Referee #2

This manuscript describes the development of approaches to induce congenital cataracts in chicken embryos in ovo. Using injection into the lens lumen of ~HH18 chicken embryos of RCAS virus constructs, the authors demonstrate that exogenous expression of the Cx50E48K and AQPOR33C mutations associated with congenital cataract in humans results in cataract formation in chicken embryos. Structural defects in the lens are described. In contrast, ectopic expression of alpha crystallin mutations associated with cataract in humans did not result in cataract in the chicken, at least at the time points analysed. However, light transmission and protein aggregation were altered, which can be forerunners of cataract development. The authors propose that these in vivo models of congenital cataract could be used to study mechanisms of cataract formation and as a tool to screen for therapeutic interventions.

The paper is well written and the data analyses are, in the main, robust. The images are of a very high standard and the figures well laid out and clearly presented. The statistical analyses seem appropriate and sufficient data is presented in the methods section to enable the work to be reproduced (although this will require referring to previously published work).

Response: We greatly appreciate the positive comments, thorough evaluation of our work, and constructive comments and suggestions.

The main limitation of the study is that it is purely descriptive. The mechanism(s) by which cataracts are induced by the mutant constructs is not established. The extent to which the manuscript advances understanding therefore is quite limited. This said, the description of the model is in itself a potentially useful advance and may be of benefit to others. Overall, given the aim of this manuscript is to disseminate information on a method for generating congenital cataracts in chicken, my feeling is that this paper may be more suitable for a specialist eye journal.

Response: Cataracts are the leading cause of blindness in the world and this study, albeit descriptive, provides a potent model for our understanding of congenital cataracts due to mutations of lens proteins and potentially for drug screening and development. In addition to the research in lens cataract formation, this model could potentially be developed as an *in vivo* platform for studying structure-function of exogenous proteins. In our study, the puncta formation in the AQP0 mutation and protein aggregation in CRYAA mutation indicate that this model could be used to study protein aggregation *in situ*. Although we have not studied other non-lens proteins, application of this model could be broad beyond lens proteins and this warrants further research and development. We have included the above in the Discussion (Page 22, lines 8-16).

Minor point:

1. The abnormal nuclear organisation that occurs would benefit from some quantification. For example, on P6, lines 7-8 it is stated that "at E4 it appeared to have more nuclei". Quantification

at different ages of the total number of nuclei, their abnormal location and % of nuclei that are aberrantly positioned would allow more robust conclusions to be drawn.

Response: As suggested by the reviewer, we have quantified nuclei numbers at E7 and E11 and included this data in the new Figure 3C.

Referee #3

The manuscript describes a well-designed study aimed at demonstrating the suitability of using chick lens expressing certain mutant proteins as a model system for studying oxidative stress-induced cataracts and their prevention or treatment by novel pharmacological compounds. The experimental methods and results are very well-described and presented.
The role of oxidative stress in lenses expressing Cx50E48K was demonstrated by their increased 4HNE staining; oxidative stress was not demonstrated in other mutants, at least in this study.

Response: We greatly appreciate the positive comments, thorough evaluation of our work, and constructive comments and suggestions.

I have a few questions/comments to be addressed by the authors:
1. What is the reason/mechanism for oxidative stress in the Cx50E48K mutant?

Response: The reviewer raised a good question regarding oxidative stress in the Cx50E48K mutant. As we described in the Discussion (page 17, line 19-23), Cx50 plays a predominant role in gap junction-mediated cell-cell communication in maintaining hemostasis of cortical fiber cells by delivering nutrients and extruding wastes. Our previously study demonstrated that mutation of Cx50E48K inhibits gap junction coupling in a dominant negative manner (Banks, Toloue et al. 2009). Therefore, the impairment of gap junction communcation will lead to accumulation of reactive oxygen species with increaesd oxidative stress, and ultimately cataract formation

2. How do we know whether the increased 4HNE is a consequence of cataract in the mutant, or whether the mutation induced an increase in 4HNE resulting in cataract? The latter would indicate that oxidative stress induced the cataract in the mutant.

Response: As the reviewer mentioned, we could not draw a definitive conclusion on the relationship between cataract formation and oxidative stress according to the current experimental results. However, as shown in Figure 4D, increased 4HNE was only observed in lens expressing the E48K mutant, indicating that the mutation is associated with oxidative stress. It is reasonable to hypothesize that oxidative stress is a causative factor for cataracts since oxidative stress has been proved to be a leading cause of cataracts both *in vivo* and *in vitro* (Ivanov, Mappes et al. 2018, Shahinfar, Keshavarzi et al. 2018).

3. Is there any specific reason for selecting 4HNE as a marker for oxidative stress?

Response: Several oxidative markers found in the body have been reported, including lipid hydroperoxides, 4-hydroxynonenal, isoprostanes (IsoPs), 8-hydroxy-2-deoxyguanosine (8-OHdG), malondialdehyde (MDA), allantoin or thiobarbituric acid reactive substances (TBARS). We selected 4-hydroxynonenal (4-HNE), a marker for lipid oxidation, to determine oxidative stress in lens since cortical lens fiber is a unique type of cells and some of the mature fiber cells cells

lack mitochondria, nuclei and other organelles. In addition, 4HNE has been generally used to detect oxidative stress in previous cataract studies both *in vivo* and *in vitro* (Babizhayev, Vishnyakova et al. 2011).

4. Testing for additional indicators of oxidative stress would strengthen the claim for its role in cataracts in the mutant lenses.

Response: We agree that inclusion of additonal inidiators would strengthen the study. As mentioned above, lens cortical fibers are a unique type of the cells and mature fibers cells lack mitochondria, nuclei and other organells. Therefore, other oxidative stress indicators including DNA's and mitochondria-based markers, are unlikely to work for lens fiber cells.

5. Do the authors expect oxidative stress to be involved in cataracts in AQP0 and crystallin mutants?

Response: Both AQP0 and crystallins are important proteins in maintaining normal lens hemeostatis and transparency. It is likely that impairment of these two protein increases oxidative stress and ultimately cataract formation. Indeed, oxidative stress is reported to be elevated in lens with AQP0 and crystallin mutations (Zhao and Yan 2018, Varadaraj and Kumari 2020) although the mutation sites are different from what we studied here.

References:

Babizhayev, M. A., K. S. Vishnyakova and Y. E. Yegorov (2011). "Telomere-dependent senescent phenotype of lens epithelial cells as a biological marker of aging and cataractogenesis: the role of oxidative stress intensity and specific mechanism of phospholipid hydroperoxide toxicity in lens and aqueous." Fundam Clin Pharmacol **25**(2): 139-162.

Banks, E. A., M. M. Toloue, Q. Shi, Z. J. Zhou, J. Liu, B. J. Nicholson and J. X. Jiang (2009). "Connexin mutation that causes dominant congenital cataracts inhibits gap junctions, but not hemichannels, in a dominant negative manner." J Cell Sci **122**(Pt 3): 378-388.

Ivanov, I. V., T. Mappes, P. Schaupp, C. Lappe and S. Wahl (2018). "Ultraviolet radiation oxidative stress affects eye health." J Biophotonics **11**(7): e201700377.

Shahinfar, J., Z. Keshavarzi, M. Ahmadi, S. Barzegar, G. Asieh and A. Abbaspour (2018). "Serum Oxidative Stress Markers in Patients with Senile Cataract and Healthy Controls." J Coll Physicians Surg Pak **28**(6): 448-451.

Varadaraj, K. and S. S. Kumari (2020). "Lens aquaporins function as peroxiporins to facilitate membrane transport of hydrogen peroxide." Biochem Biophys Res Commun **524**(4): 1025-1029.

Zhao, W.-J. and Y.-B. Yan (2018). "Increasing susceptibility to oxidative stress by cataract-causing crystallin mutations." International Journal of Biological Macromolecules **108**: 665-673.

REVIEWERS' COMMENTS:

Reviewer #1 (Remarks to the Author):

The authors adequately addressed this reviewer's comments.

Reviewer #2 (Remarks to the Author):

In this revised manuscript the authors have made a number of changes in response to the reviewers' comments that have helped strengthen the manuscript. This includes some new analyses as well as clarification of detail within the text. I have only a few minor points:

1. The authors have quantified the number of nuclei in lens injected with the Cx50E48K mutation (new data in Figure 3C). However, this new analysis is not mentioned in the text.

2. New text P19, lines 15-21. I am not convinced by the argument that because the chicken lifespan is shorter than in human this means that the majority of cataracts may appear at embryonic stages in chicken. Rather than life span, a better, and more relevant comparison would be of the time scale of lens development in chicken versus human. In particular the events that occur pre versus post hatching/birth.

Reviewer #1 (Remarks to the Author):

The authors adequately addressed this reviewer's comments.

Response: I'd appreciate the comment and review of our paper.

Reviewer #2 (Remarks to the Author):

In this revised manuscript the authors have made a number of changes in response to the reviewers' comments that have helped strengthen the manuscript. This includes some new analyses as well as clarification of detail within the text. I have only a few minor points:

1. The authors have quantified the number of nuclei in lens injected with the Cx50E48K mutation (new data in Figure 3C). However, this new analysis is not mentioned in the text.

Response: We have included the description of new analysis in the Results (Page 6, lines 10-12).

2. New text P19, lines 15-21. I am not convinced by the argument that because the chicken lifespan is shorter than in human this means that the majority of cataracts may appear at embryonic stages in chicken. Rather than life span, a better, and more relevant comparison would be of the time scale of lens development in chicken versus human. In particular the events that occur pre versus post hatching/birth.

Response: The reviewer raised a good point for the cataract formation during chicken lens development. The major process of lens development includes induction (not relevant here), morphogenesis, differentiation and growth. For morphological changes and differentiation, the major course contains epithelial cell proliferation, epithelial differentiation, fiber cell proliferation, fiber cell elongation and organelle degeneration. In fact, all the processes described above could be seen throughout whole life span of human as lens continuously grows in human. For chicken, lens consisted of epithelial cell and primary fiber mass at embryonic day (E) 3. By E12, both primary fiber cell and secondary fiber cell could be seen in chicken lens (Bassnett and Winzenburger, 2003). At the same time organelle degeneration could be observed (Bassnett and Beebe, 1992). By the time of hatching, the organelle-free lens region expands to match the diameter of the pupil. For growth rate, chick lens continues to grow up to 34 weeks after which the lens grows slowly (Priolo et al., 1999). Due to the early differentiation, organelle degeneration and denucleation of central lens fibers, the embryonic chick lens at late developmental stage exhibits characteristics of lens as seen in postnatal human lens. We have revised the statement in the text (Page 14, lines 7-9).

Bassnett, S., and D.C. Beebe. 1992. Coincident loss of mitochondria and nuclei during lens fiber cell differentiation. *Developmental dynamics : an official publication of the American Association of Anatomists*. 194:85-93.

Bassnett, S., and P.A. Winzenburger. 2003. Morphometric analysis of fibre cell growth in the developing chicken lens. *Exp Eye Res*. 76:291-302.

Priolo, S., J.G. Sivak, and J.R. Kuszak. 1999. Effect of age on the morphology and optical quality of the avian crystalline lens. *Exp Eye Res*. 69:629-640.
